# Ensilication Improves the Thermal Stability of the Tuberculosis Antigen Ag85b and an Sbi-Ag85b Vaccine Conjugate

**DOI:** 10.1038/s41598-019-47657-9

**Published:** 2019-08-08

**Authors:** A. A. Wahid, A. Doekhie, A. Sartbaeva, J. M. H van den Elsen

**Affiliations:** 1 Department of Biology and Biochemistry, University of Bath, Bath, UK; 2 Department of Chemistry, University of Bath, Bath, UK

**Keywords:** Conjugate vaccines, Tuberculosis

## Abstract

There is an urgent need for the development of vaccine thermostabilisation methodologies as the maintenance of a continuous and reliable cold chain remains a major hurdle to the global distribution of safe and effective vaccines. Ensilication, a method that encases proteins in a resistant silica cage has been shown to physically prevent the thermal denaturation of a number of model proteins. In this study we investigate the utility of this promising approach in improving the thermal stability of antigens and vaccine conjugates highly relevant to the development of candidate tuberculosis vaccines, including antigen 85b conjugated with the *Staphylococcus aureus*-protein based adjuvant Sbi. Here we analyse the sensitivity of these constructs to thermal denaturation and demonstrate for the first time the benefits of ensilication in conferring these vaccine-relevant proteins with protection against temperature-induced loss of structure and function without the need for refrigeration. Our results reveal the potential of ensilication in facilitating the storage and transport of vaccines at ambient temperatures in the future and therefore in delivering life-saving vaccines globally, and in particular to remote areas of developing countries where disease rates are often highest.

## Introduction

In order to prevent thermal denaturation and loss of potency, the vast majority of current vaccines necessitate storage and transportation at refrigerated temperatures (2–8 °C). However, a lack of infrastructure, equipment and effective logistics required to support this cold chain, particularly in developing countries, can lead to 75–100% of vaccines being exposed to suboptimal temperatures during dissemination. As a consequence, up to 50% of manufactured vaccines are discarded before administration^1,2^. The cold chain can also raise vaccination costs by up to 80%^2^, further limiting the widespread distribution of safe and effective vaccines.

Lyophilisation (freeze-drying) has been shown to improve the thermal stability a number of routinely-used vaccines, particularly live-attenuated varieties such as Bacille Calmette-Guérin (BCG) and measles, mumps and rubella (MMR), by dramatically retarding degradative processes^3,4^. Although protectants and stabilising agents are used to prevent damage during lyophilisation, which further raises vaccination costs, the combined stress of harsh freezing and drying makes this stabilisation method less suitable for subunit or conjugate vaccines composed primarily of individual proteins^3,5^.

Thus, given the barriers associated with the maintenance of a continuous and reliable cold chain, and lyophilisation, research on novel methods to thermally stabilise vaccines has risen substantially in the last decade. Thermostabilisation approaches in development include alternative drying formats such as spray, spray-freeze and vacuum-foam drying^4^, the use of sugar membranes^6–8^, lipids, emulsions, natural and synthetic polymers^9–11^, peptide engineering^12,13^ and ensilication^14^.

Ensilication, a method recently developed by our group, is based on the solution-gelation process whereby negatively-charged silanol groups associate with charged amino acid residues through non-covalent electrostatic interactions^14^. This results in growth of a protective silica cage around protein molecules^14,15^ which is subsequently vacuum-filtered and dried. The resultant silica-coated powder is capable of physically preventing thermal denaturation of the encased immobilised proteins. Subsequent chemical digestion of the silica is then used to release the protein back into solution in a native state suitable for applications such as vaccines  and other therapeutic treatments. This technique has been shown to confer the model protein hen egg white lysozyme (HEWL) with resistance against boiling at 100 °C for 5 hours as well as aging at room temperature for over 6 months^14^. Similar stabilisation of haemoglobin and tetanus toxin C-fragment (TTCF) has also been demonstrated.

In this study we aimed to evaluate the use of ensilication in improving the thermal stability of the *Mycobacterium tuberculosis* antigen 85b (Ag85b); a leading candidate in the development of new tuberculosis (TB) vaccines, and a novel Sbi III-IV-Ag85b vaccine conjugate. Ag85b is an abundant extracellular fibronectin-binding protein functioning as a mycolyl transferase involved in cell wall biosynthesis. It is highly immunogenic, possesses several immunodominant T cell epitopes and induces specific cell-mediated and humoural immune responses in infected patients^16,17^. However, Ag85b alone is not sufficiently immunogenic to generate a protective immune response against *M*. *tuberculosis*. Therefore, potential Ag85b-containing vaccines being investigated are viral vector-based^18,19^, contain other tuberculosis antigens including ESAT-6^20–23^ and TB 10.4^24^ and/or incorporate adjuvants such as toll-like receptor agonists (IC31)^25–31^ and liposomes (CAF01)^32–38^. In this regard, our group has recently revealed that domains III and IV of the *Staphylococcus aureus* immunomodulator Sbi can enhance the immunogenicity of Ag85b. In normal human serum, Ag85b co-incubated with or conjugated to Sbi III-IV is rapidly opsonised by activation products of the central complement component C3, including the natural molecular adjuvant C3d, which can lead to C3d-complement receptor 2 (CR2) and antigen-B cell receptor (BCR) co-ligation *in vivo*. This effect is translated into the generation of a more rapid and raised anti-Ag85b IgG titres compared to Ag85b alone^39^ by lowering the threshold of antigen required for B cell activation (as described in:^40–42^). In addition, antigen-presentation by the activated B cells and follicular dendritic cells can promote T cell responses^43^ and the generation of memory B cells, respectively^44–46^.

The limited data available on the stability of Ag85b reveal that this antigen is inherently thermally unstable. Using circular dichroism spectroscopy, Shi and colleagues^47^ have shown Ag85b undergoes an irreversible loss of secondary structure at high temperatures and possesses a melting temperature of 73.7 °C. In addition, although the functionality of Sbi III-IV as an adjuvant compound has been investigated, its stability and temperature sensitivity, on its own or as part of a vaccine conjugate with Ag85b, remains unexplored.

 Following  on from our previous research using model proteins, here we demonstrate the application of ensilication in improving the thermal stability of proteins in a vaccine setting for the first time. We perform an in-depth analysis of the thermal instability of the model TB antigen Ag85b and show that ensilication protects this antigenic protein, pivotal in the design of current and future candidate TB vaccines, against thermal degradation, aggregation and unfolding. We also bridge the gap in knowledge surrounding the thermal sensitivity of the novel Sbi III-IV-Ag85b TB vaccine conjugate and Sbi III-IV vaccine adjuvant and provide strong evidence that ensilication enhances the thermal resistance of these vaccine proteins. Overall, we provide crucial support for the use of ensilication as a method for thermostabilising vaccine-relevant proteins without the need for lyophilisation or refrigeration.

## Results

**Figure 1:**
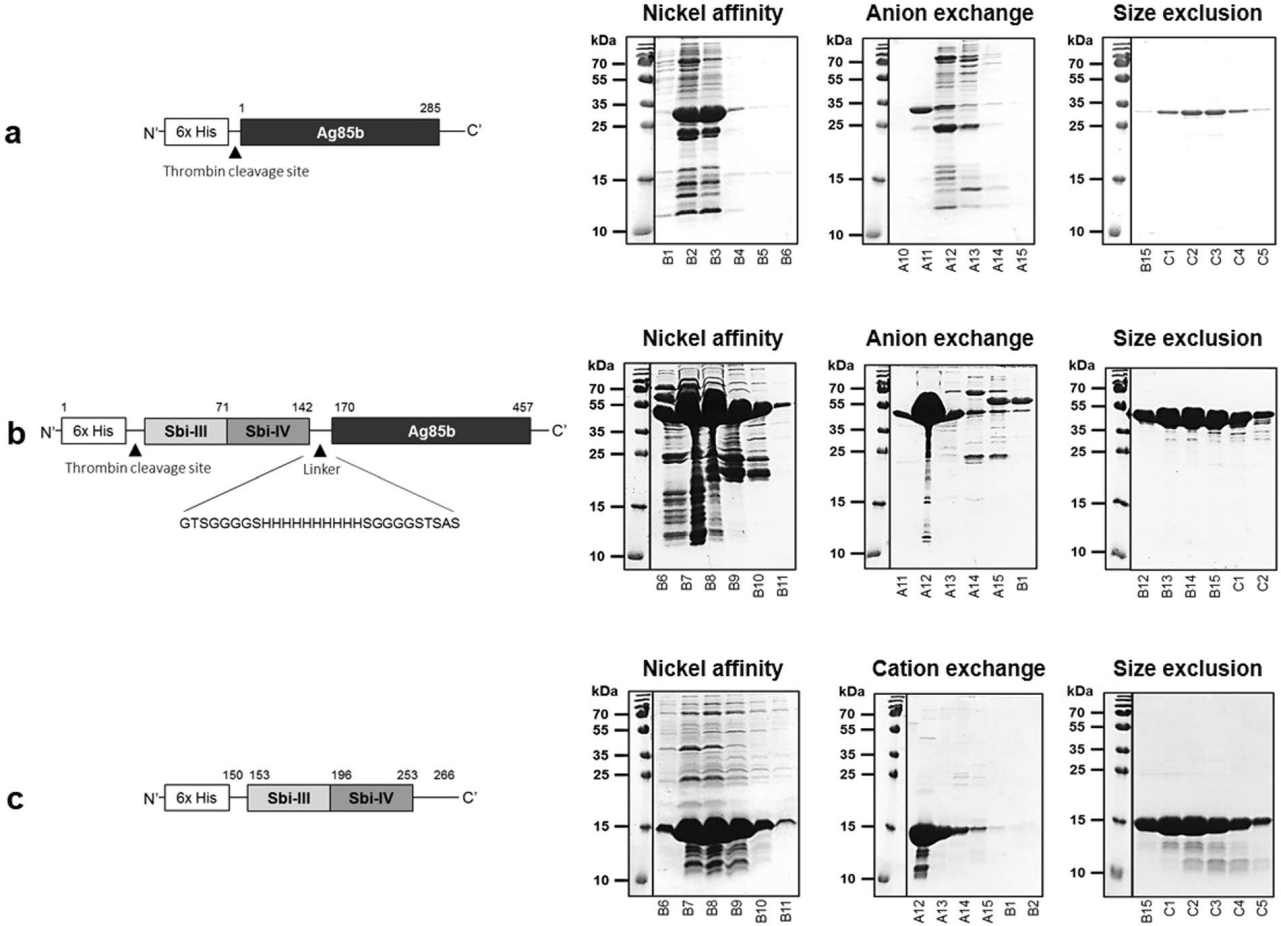
Design and purification of the Ag85b (**a**), Sbi III-IV-Ag85b (**b**) and Sbi III-IV (**c**) constructs. Schematic representations of the constructs are shown on the left and SDS-PAGE profiles of the nickel affinity, ion exchange and size exclusion chromatography elution fractions are shown in the right panels. All three constructs bear an N-terminal 6x His-tag and the numbering of Ag85b and Sbi III-IV amino acid residues is according to their respective full-length proteins. The Sbi III-IV-Ag85b conjugate contains Sbi domains III and IV conjugated via a 28 amino acid histidine and glycine-rich linker to Ag85b at the C-terminus. Note: the yield of Ag85b shown is from 1 L of culture while the yield from Sbi III-IV-Ag85b and Sbi III-IV is from 4 L of culture. Details of the protein physiochemical properties can be found in Supplementary Table S1. Supplementary Fig. S1 includes purification chromatogram profiles. Full-length gel images of (**a**–**c**) are included in Supplementary Figs S11–S13.

In order to gain a better understanding of the temperature sensitivity of Ag85b, the Sbi III-IV-Ag85b vaccine conjugate and Sbi III-IV adjuvant, and the utility of ensilication in improving their thermal stability, these vaccine-relevant proteins were recombinantly expressed and purified (Fig. 1, Supplementary Table S1, Fig. S1), ensilicated and subjected to harsh short-term conditions of thermal denaturation or longer-term aging in the form of an ensilicated powder. The vaccine proteins were then released from the silica cage back into solution and ensilication-mediated protection against temperature-induced loss of structural integrity and function was subsequently analysed by comparison to solutions of native protein.

### Antigen 85b is sensitive to thermal denaturation

**Figure 2:**
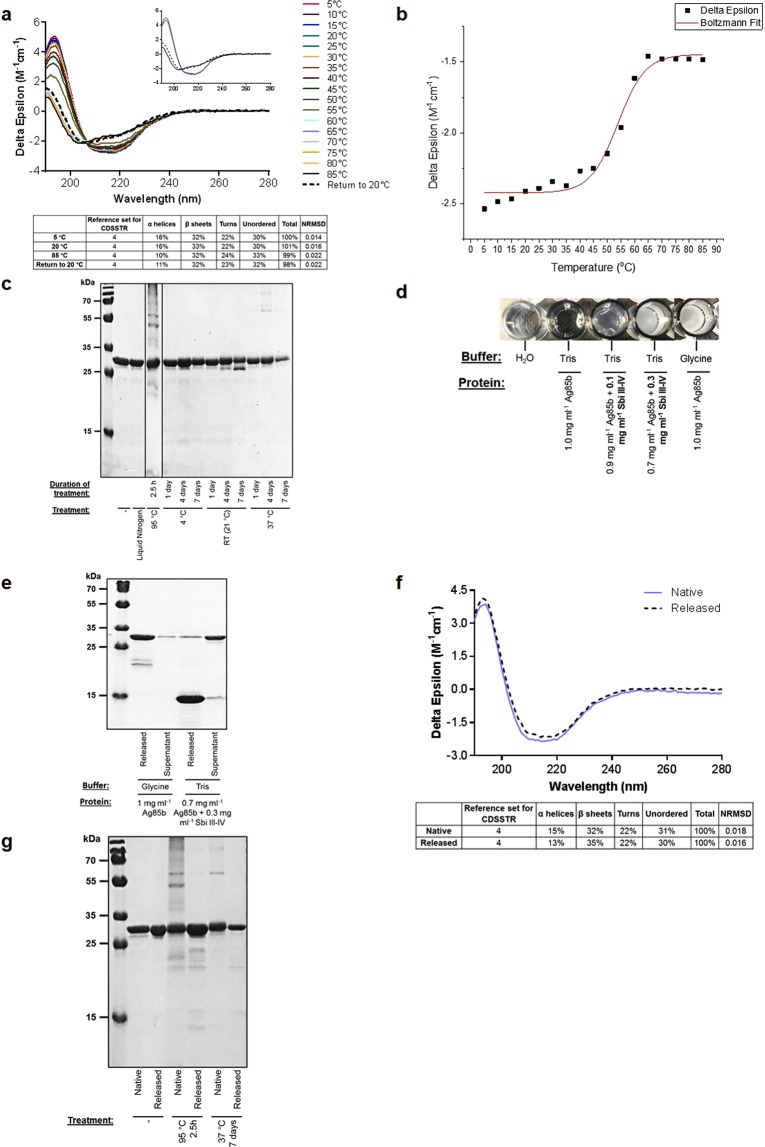
Ensilication improves the thermal stability of Ag85b. (**a**) Thermal melt circular dichroism (CD) spectra showing irreversible thermal unfolding of Ag85b. CD spectra at 5 °C, 20 °C, 85 °C and upon return to 20 °C are displayed in the inset figure. Deconvolution was performed using DichroWeb. (**b**) Boltzmann sigmoidal fit of CD thermal denaturation curve at 222 nm depicting a melting temperature of 53.9 °C. (**c**) Temperature-induced loss of Ag85b structural integrity visualised by SDS-PAGE. (**d**) Ag85b ensilication trials for visualisation of sol-gel precipitates showing 50 mM glycine at pH 7.4 yields the highest precipitation. Water was included as a negative control. (**e**) SDS-PAGE analysis illustrating the composition of the released solution and unensilicated supernatant following ensilication of Ag85b in the glycine buffer or presence of Sbi III-IV. See Supplementary Table S2 for protein concentrations. (**f**) CD spectroscopy results depicting secondary structural comparability of native and released Ag85b. Spectra are representative of 3 replicates. Deconvolution was performed using DichroWeb. (**g**) SDS-PAGE illustrating thermal degradation and aggregation of native but not released Ag85b. Particle analysis and CD of thermally-treated native and released Ag85b can be found in Supplementary Figs S2 and S3 respectively. Full-length gel images of (**c**,**e**,**g**) are included in Supplementary Figs S14–S16.

To gain a more in-depth understanding of the thermal stability of Ag85b, changes in secondary structure induced by thermal denaturation were monitored using circular dichroism (CD). As illustrated in Fig. 2a an increase in temperature from 5 °C to 65 °C causes a considerable loss of secondary structure and at 85 °C the antigenic protein has lost 37.5% of its helicity and gained a small percentage of turns and unordered secondary structures. On returning to 20 °C, Ag85b does not show any clear signs of refolding indicating irreversible thermal denaturation, consistent with the observed precipitate formation. A sigmoidal fit of the thermal denaturation curve at 222 nm reveals this Ag85b construct has a melting temperature of 53.9 °C (Fig. 2b).

Further analysis of temperature sensitivity showed that Ag85b exhibits the formation of visible precipitation as well as clear signs of aggregation and degradation when exposed to 95 °C for 2.5 hours (Fig. 2c, Supplementary Fig. S2). The protein also loses its structural integrity after 4 days at room temperature (21 °C), and unfolds (Supplementary Fig. S3) and forms higher molecular weight aggregates at 37 °C for 4 or more days (Fig. 2c, Supplementary Fig. S2). On the other hand, flash freezing in liquid nitrogen and storage at 4 °C does not appear to affect the structural integrity of the antigen.

### Ensilication confers Ag85b with resistance to heat-induced changes in structural integrity

As the isoelectric point (pI) of our Ag85b construct (pI: 5.65) is lower than the proteins previously ensilicated (horse haemoglobin: 6.5, HEWL: 9.4)^14^, ensilication of Ag85b was initially trialled on a small scale under different conditions. As illustrated in Fig. 2d, ensilication of the antigen in the standard 50 mM Tris pH 7 buffer used previously^14^, fails to produce any visible ensilicated protein precipitates. Addition of Sbi III-IV improves the amount of sol-gel precipitate formation in a concentration-dependent manner (Fig. 2d) although a moderate amount of Ag85b is left unensilicated (Fig. 2e, Supplementary Table S2). On the other hand, the 50 mM glycine pH 7.4 buffer produces the most optimal results, yielding the greatest precipitation (Fig. 2d) which is reflected by the presence of only a small amount of protein appearing in the unensilicated supernatant (Fig. 2e, Supplementary Table S2). Although a few degradation fragments are visible, the majority of the released protein remains intact.

On the basis of these findings, a larger scale ensilication of Ag85b in 50 mM glycine pH 7.4 was performed as per the method established  previously^14^ which resulted in the production of 9 mg of a dry white powder composed of silica-encased Ag85b from 3.5 mg of protein. The unensilicated flowthrough did not contain a measurable concentration of protein suggesting that all of the starting material was ensilicated. Ag85b was subsequently released from silica back into solution using an acidic sodium fluoride solution as described previously^14^. On average, release of 1 mg of ensilicated powder yielded 0.27 mg of protein in 1 ml of solution. Comparison of the CD spectra of native and released Ag85b showed negligible differences between the secondary structures of the two proteins (Fig. 2f). To analyse thermal stability, ensilicated Ag85b was subsequently subjected to conditions of thermal denaturation after which the protein was released from the powder and compared to native unensilicated Ag85b treated in the same way, using CD, particle analysis and SDS-PAGE. As is evident from the results shown in Fig. 2g and Supplementary Fig. S2, the formation of insoluble aggregates following incubation at 95 °C for 2.5 hours and 37 °C for 7 days is visible in the native but not in the released samples. Although there are some signs of degradation in both of the thermally-treated released samples, overall, the released proteins retain a greater degree of structural integrity compared to their native counterparts. Importantly, ensilicated Ag85b released following 7 days at 37 °C retains a similar secondary structure to native untreated Ag85b whereas native Ag85b shows clear signs of unfolding when treated in the same way (Supplementary Fig. S3).

### High temperatures and longer-term storage cause degradation and aggregation of Sbi III-IV-Ag85b

**Figure 3:**
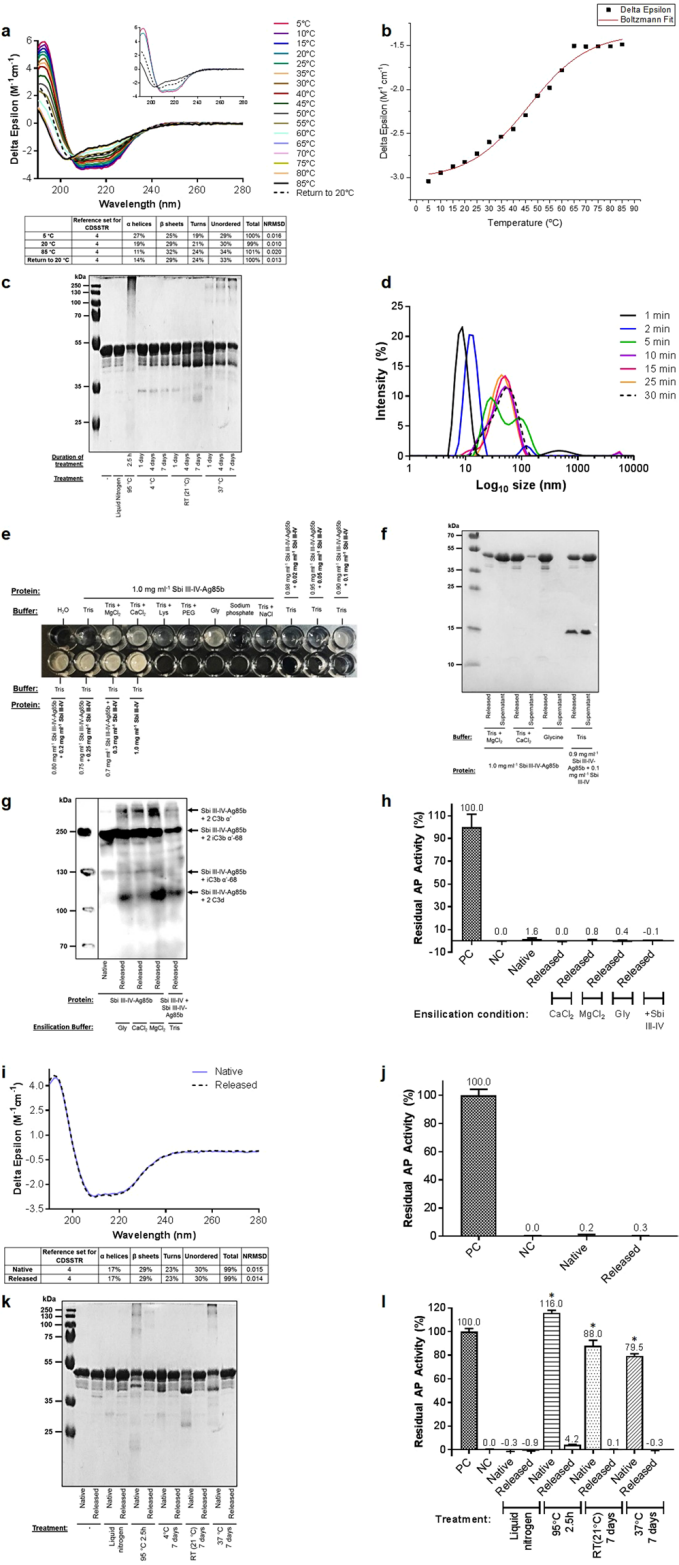
Ensilication protects Sbi III-IV-Ag85b against thermal denaturation. (**a**) CD spectra showing partially reversible thermal unfolding of Sbi III-IV-Ag85b. CD spectra at 5 °C, 20 °C, 85 °C and upon return to 20 °C are displayed in the inset figure. Deconvolution was performed using DichroWeb. (**b**) Boltzmann sigmoidal fit of CD thermal denaturation curve at 222 nm indicating a melting temperature of 46.3 °C. (**c**) Temperature-induced loss of Sbi III-IV-Ag85b structural integrity visualised by SDS-PAGE. (**d**) Time-course dynamic light scattering size distribution by intensity plot of Sbi III-IV-Ag85b ensilication in 50 mM Tris pH 7 showing a maximal particle size of 55 nm. (**e**) Sbi III-IV-Ag85b ensilication trials for visualisation of sol-gel precipitates showing that MgCl_2_, CaCl_2_, glycine and Sbi III-IV improve precipitate formation. Water and Sbi III-IV alone were included as negative and positive controls respectively. (**f**) SDS-PAGE analysis showing protein levels in the released solution and unensilicated supernatant following ensilication of Sbi III-IV-Ag85b under the four conditions depicted. See Supplementary Table S3 for protein concentrations. (**g**) Anti-Sbi western blot analysis of C3 activation in normal human serum following incubation with native or released Sbi III-IV-Ag85b. Higher molecular weight bands depicting opsonisation of native and released Sbi III-IV-Ag85b with C3 activation products are evident. All samples are at a concentration of 27 μM except for the released sample containing Sbi III-IV which is at 18 μM. (**h**) Alternative pathway activity analysis showing comparable complement depletion activity for native Sbi III-IV-Ag85b (4 µM) and Sbi III-IV-Ag85b (4 µM) released following ensilication under the conditions shown. Data are displayed as mean values (n = ≥ 2) ± standard deviation from the mean. A one-way ANOVA with Dunnett’s multiple comparisons analysis confirmed no significant differences in activity between native Sbi III-IV-Ag85b and the released Sbi III-IV-Ag85b samples (*P* > 0.05). (**i**) CD spectroscopy showing the near-identical secondary structures of native and released Sbi III-IV-Ag85b. Spectra are representative of 3 replicates. Deconvolution was performed using DichroWeb. (**j**) Alternative pathway assay depicting comparable functional activities for native Sbi III-IV-Ag85b (4 μM) and Sbi III-IV-Ag85b (4 µM) released subsequent to ensilication in 50 mM glycine pH 7.4. Data are displayed as mean values (n = ≥ 2) ± standard deviation from the mean. An unpaired t-test confirmed no significant difference in activity between native and released Sbi III-IV-Ag85b (*P* > 0.05). (**k**) SDS-PAGE profile illustrating thermal denaturation and aggregation in native but not released Sbi III-IV-Ag85b samples. (**l**) Alternative pathway activity analysis displaying retention of complement depletion activity in released but not in native thermally-treated Sbi III-IV-Ag85b (4 μM). Data are displayed as mean values (n = ≥ 2) ± standard deviation from the mean. A one-way ANOVA with Dunnett’s multiple comparisons analysis was used to analyse differences in activity compared to native untreated Sbi III-IV-Ag85b. *P < 0.01. DLS, CD and opsonisation analysis of thermally-treated native and released Sbi III-IV-Ag85b can be found in Supplementary Figs S4–S6, respectively. Full-length gel images of (**c**,**f**,**k**) are included in Supplementary Figs S17–S19 and full-length western blot image of (**g**) is shown in Supplementary Fig. S20.

We next aimed to analyse the thermal stability of a construct comprised of Sbi III-IV tethered to Ag85b which has shown promise as a novel TB vaccine conjugate. Sbi III-IV promotes opsonisation of Ag85b with C3 activation products leading to C3d-CR2 antigen-BCR co-ligation and an enhanced humoural immune response *in vivo*^39^. Performance of a thermal melt CD spectroscopic study showed that the conjugate construct, composed primarily of alpha-helices and beta-sheets, displays changes in CD signal from 5 °C to 65 °C indicative of a loss of secondary structure (Fig. 3a) and has a melting temperature of 46.3 °C (Fig. 3b). On a return to 20 °C Sbi III-IV-Ag85b is able to recover a small degree of the 59% helicity lost at 85 °C but is incapable of fully refolding and regaining its native conformation, consistent with the visible precipitates observed.

Furthermore, the conjugate shows clear signs of precipitation and non-native aggregation following exposure to 95 °C for 2.5 hours and 37 °C after 1 day as well as a loss of structural integrity after 4 days at 21 °C (Fig. 3c, Supplementary Fig. S4). Secondary structure analysis also shows that the construct unfolds and loses helicity following the 95 °C and 37 °C treatments (Supplementary Fig. S5). For the most part the integrity of the construct appears to be unaffected by the liquid nitrogen treatment and storage at 4 °C.

### Small scale trials reveal optimal buffer conditions for Sbi III-IV-Ag85b ensilication

Initial Sbi III-IV-Ag85b (pI: 6.53) ensilication attempts using the standard protocol showed some evidence of particulate formation but failed to produce a powder as almost all of the protein appeared in the unensilicated filtration flowthrough. Dynamic light scattering analysis of ensilication of the conjugate shows a time-dependent modest increase in particle size from 9 nm after 1 minute to a maximum of 55 nm  after 10 minutes (Fig. 3d). This lack of large (>200 μm) silica-deposited protein particles, which would subsequently form dense aggregated networks, could explain why the ensilicated Sbi III-IV-Ag85b protein passed through the filter (0.7 μm pore size) and no powder could be acquired.

Small scale trials under different conditions were subsequently performed in an attempt to optimise Sbi III-IV-Ag85b ensilication. Improvements in the amount of precipitate formation were evident in 50 mM Tris pH 7 supplemented with 20 mM calcium chloride or 20 mM magnesium chloride, in 50 mM glycine pH 7.4, and in a concentration-dependent manner with the addition of Sbi III-IV (0.02–0.3 mg ml^−1^) (Fig. 3e).

Small amounts of Sbi III-IV-Ag85b powder were produced following ensilication under these conditions and the protein was subsequently released back into solution (Fig. 3f). Presence of residual protein in the unensilicated supernatant under all the conditions except glycine suggests all of the starting protein is incorporated into the silica matrix when ensilication is performed in 50 mM glycine pH 7.4 (Supplementary Table S3). In the presence of magnesium chloride the majority of Sbi III-IV-Ag85b is left unensilicated while the inverse is true in the presence of calcium chloride (Fig. 3f). Although with the addition of Sbi III-IV approximately 60% of the protein solution appears in the ensilication supernatant (Supplementary Table S3), both Sbi III-IV and Sbi III-IV-Ag85b are ensilicated and released. Importantly, despite the variations in ensilication efficiency, the functional activity of Sbi III-IV-Ag85b released following ensilication under the four conditions examined is equivalent to the untreated conjugate in terms of its ability to induce opsonisation via C3 activation (Fig. 3g) and deplete serum C3 levels through alternative pathway (AP) activation (Fig. 3h).

### Ensilication prevents heat-induced loss of Sbi III-IV-Ag85b structure and function

Similar to Ag85b, 50 mM glycine pH 7.4 appeared to be the most optimal ensilication buffer for Sbi III-IV-Ag85b, and a larger scale ensilication reaction under these conditions was performed, yielding 15 mg of powder from 5.5 mg of protein. The unensilicated filtration flowthrough did not contain a measurable amount of protein and on average, release of 1 mg of ensilicated powder yielded 0.52 mg of protein in 1 ml of solution. The CD spectra of native and released Sbi III-IV-Ag85b were found to overlay almost completely, indicating that the secondary structure of the conjugate released from silica is near-identical to its native equivalent (Fig. 3i). Moreover, consistent with Fig. 3h, Sbi III-IV-Ag85b released from the ensilicated powder is functionally comparable to the native unensilicated protein in terms of AP activation-mediated C3 depletion (Fig. 3j).

In order to determine whether ensilication could improve the thermal stability of this conjugate, both a native solution and ensilicated powder of Sbi III-IV-Ag85b were subjected to the same conditions of thermal denaturation as the native conjugate, shown in Fig. 3c, after which the ensilicated protein was released back into solution. As depicted in Fig. 3k and Supplementary Figs S4 and S5 the formation of insoluble aggregates as well as signs of unfolding and loss of helicity is apparent in the native but not the released samples following incubation at 95 °C for 2.5 hours and 37 °C for 7 days. Protein degradation at 21 °C is also only visible in the native samples. In contrast, no clear differences between the native and released protein are observable when exposed to the liquid nitrogen treatment or 4 °C for 7 days.

In terms of functionality, in comparison to the native construct, ensilicated and released Sbi III-IV-Ag85b retains the majority of its functional activity when exposed to conditions of thermal denaturation (Fig. 3l). This is with the exception of the liquid nitrogen treatment where both the native and released samples are fully functional. A small decline in ability to induce AP activation-mediated C3 depletion of the released protein after 95 °C for 2.5 hours can be seen but this is insignificant in comparison to its native counterpart. Importantly, opsonisation of the ensilicated samples released following the 95 °C, 21 °C and 37 °C treatments with the C3 activation products iC3b α′-68 and C3d is also apparent (Supplementary Fig. S6), providing further evidence that ensilication facilitates the retention of Sbi III-IV-Ag85b funtionality under conditions of thermal denaturation.

### Sbi III-IV undergoes reversible thermal unfolding but a loss of structural integrity and function during longer-term storage

**Figure 4:**
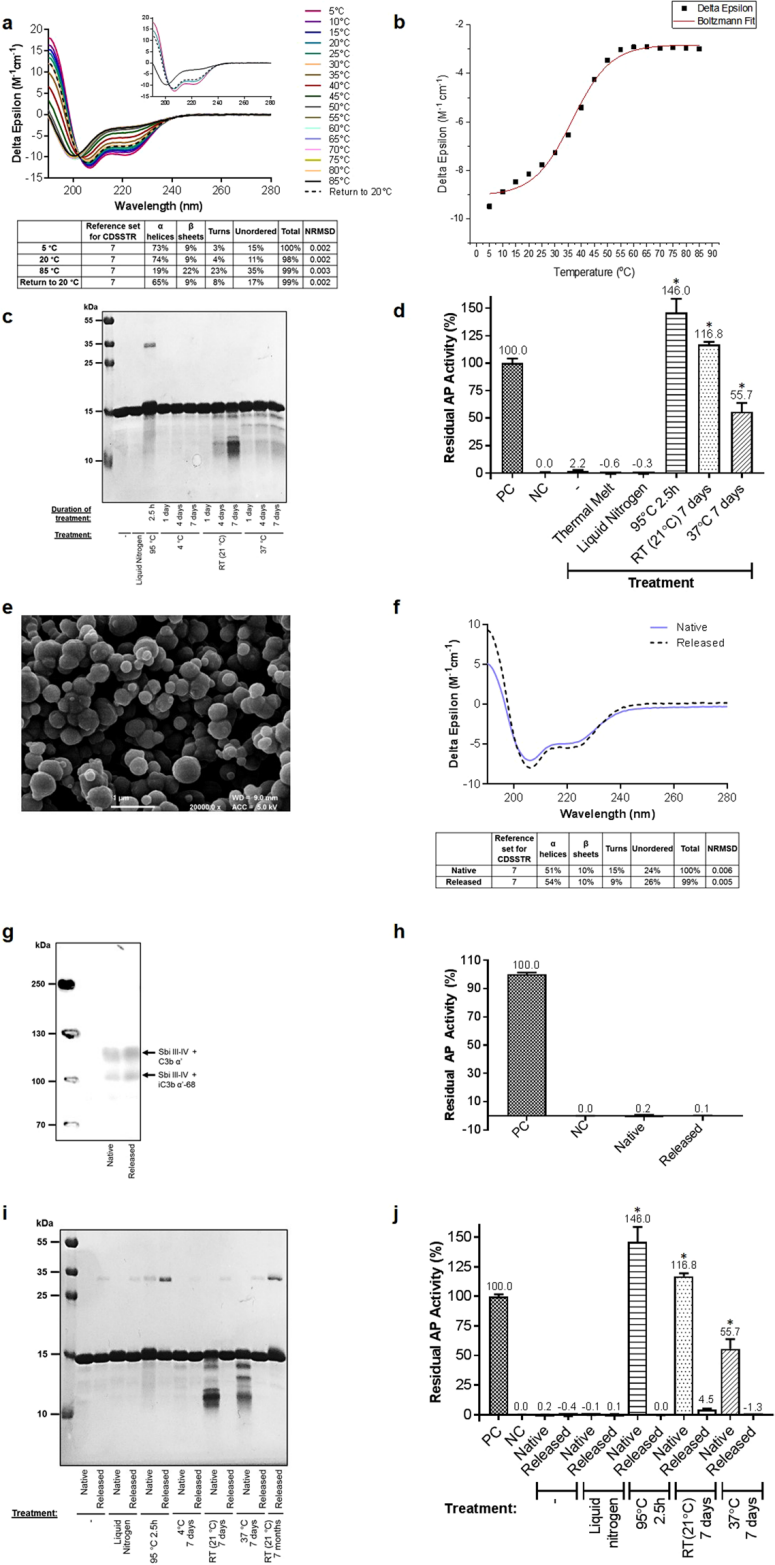
Thermostabilisation of Sbi III-IV using ensilication. (**a**) Thermal melt circular dichroism spectroscopic study showing reversible thermal unfolding of Sbi III-IV. CD spectra at 5 °C, 20 °C, 85 °C and upon return to 20 °C are displayed in the inset figure. Deconvolution of CD data was performed using DichroWeb. (**b**) Boltzmann sigmoidal fit of CD thermal denaturation curve at 222 nm depicting a melting temperature of 35.6 °C. (**c**) Temperature-induced loss of Sbi III-IV structural integrity visualised by SDS-PAGE. (**d**) Thermal denaturation-mediated loss of Sbi III-IV (0.5 μM) complement depletion activity demonstrated by alternative pathway activity analysis. Data are displayed as mean values (n = ≥ 2) ± standard deviation from the mean. A one-way ANOVA with Dunnett’s multiple comparisons analysis was used to analyse differences in activity compared to native untreated Sbi III-IV., *P < 0.01. (**e**) FESEM image of ensilicated Sbi III-IV powder at 20,000x magnification. Scale bar is shown at the bottom of the image. See Supplementary Fig. S7 for FESEM images at 650x and 5,000x magnification. (**f**) CD spectra showing similar secondary structures for native and released Sbi III-IV. Spectra are representative of 10 replicates. Deconvolution of CD data was performed using DichroWeb. (**g**) Anti-Sbi western blot analysis illustrating comparable C3 activation by native and released Sbi III-IV (both at 27 μM). (**h**) Alternative pathway activity analysis demonstrating comparable C3 depletion activities for native and released Sbi III-IV (both at 0.5 μM). Data are displayed as mean values (n = ≥ 2) ± standard deviation from the mean. An unpaired t-test confirmed no significant difference in activity between native and released Sbi III-IV (*P* > 0.05). (**i**) SDS-PAGE illustrating thermal degradation of native but not released Sbi III-IV. A small amount of dimeric Sbi III-IV is present in all released samples. (**j**) Alternative pathway activity analysis showing temperature-induced loss of function in native but not in released Sbi III-IV (0.5 μM) samples. Data are displayed as mean values (n = ≥ 2) ± standard deviation from the mean. A one-way ANOVA with Dunnett’s multiple comparisons analysis was used to analyse differences in activity compared to native untreated Sbi III-IV. *P < 0.01. DLS, CD and opsonisation analysis of thermally-treated native and released Sbi III-IV can be found in Supplementary Figs S8–S10, respectively. Full-length gel images of (**c**,**i**) are included in Supplementary Figs S21 and S22. Full-length western blot image of (**g**) is shown in Supplementary Fig. S20.

As we have previously shown, conjugation is not required for Sbi III-IV to fulfil its adjuvant role and enhance the immunogenicity of Ag85b^39^. Hence, we also endeavoured to characterise the thermal sensitivity of Sbi III-IV on its own. A thermal melt CD spectroscopic study (Fig. 4a) showed that Sbi III-IV, which is largely alpha-helical, displays a substantial loss of secondary structure during a temperature ramp from 5 °C to 55 °C and has a relatively low melting temperature of 35.6 °C (Fig. 4a,b). At 85 °C, Sbi III-IV has lost close to 75% of its helicity and gained some beta-sheets, turns and unordered secondary structures. However, upon returning to 20 °C, the construct refolds completely, producing a CD spectrum that is very similar to that of its native state.

Additional thermal stability experiments with Sbi III-IV, depicted in Fig. 4c,d, demonstrated that the protein remains unaffected by the liquid nitrogen freeze-thawing treatment (indicated by the presence of a single protein band and functional activity comparable to untreated Sbi III-IV). Full functional activity is also retained following the reversible thermal unfolding observed in the CD analysis. However, heating at 95 °C causes the appearance of a protein smear and formation of a dimer at approximately double the molecular weight of Sbi III-IV (30 kDa), coinciding with complete loss of function. In addition, storage at room temperature (21 °C) and 37 °C results in the generation of low molecular weight degradation products and loss of activity. As depicted in Supplementary Figs S8 and S9, the 95 °C and 37 °C treatments also cause an increase in Sbi III-IV polydispersity as well as unfolding and loss of helicity.

### Ensilication protects Sbi III-IV against thermal denaturation

Now that we had established that Sbi III-IV is thermally unstable, we aimed to examine whether ensilication could improve its thermal stability. On average, 10 mg of Sbi III-IV (pI: 9.27) produced 35 mg of powder, which Field Emission Scanning Electron Microscopy (FESEM) revealed is composed of a dense network of spherical particles of varying sizes (<0.25 um to >0.625 um) (Fig. 4e, Supplementary Fig. S7). 100% of the starting material was successfully ensilicated and on average, 1 mg of ensilicated powder yielded 0.27 mg of protein in 1 ml of solution. As depicted in Fig. 4f, the CD spectra of native and released Sbi III-IV are very similar, with the largest differences observed being in the fraction of β-sheets. Importantly, released Sbi III-IV is functionally comparable to its native equivalent in terms of promoting opsonisation of the protein with C3 activation products (Fig. 4g) via AP activation-mediated consumption of C3 (Fig. 4h).

We next subjected the ensilicated Sbi III-IV powder to the same thermal denaturation conditions as the native protein in Fig. 4c. As demonstrated in Fig. 4i and Supplementary Figs S8 and S9, the appearance of the protein smear after 2.5 hours at 95 °C and lower molecular weight degradation fragments induced by incubation at 21 °C and 37 °C (and to a lesser extent at 4 °C) as well as an increase in polydispersity, unfolding and loss of helicity was visible in the native but not in the released samples. In addition, the ensilicated sample released after 7 months at 21 °C showed almost no signs of breakdown while significant degradation was observed in its native complement incubated at the same temperature for a substantially shorter period of 7 days. Interestingly, a small amount of a higher molecular weight product at the size of dimeric Sbi III-IV was present in all the released samples.

With regards to functional activity, while native Sbi III-IV loses most, if not all, of its functionality, the ability of thermally-treated released Sbi III-IV to cause AP activation-mediated C3 consumption is almost equivalent to untreated native Sbi III-IV (Fig. 4j). Although there is a small reduction in activity of the released protein at 21 °C for 7 days, this is insignificant when compared to its native counterpart. Importantly, opsonisation of the ensilicated samples released following the 95 °C, 21 °C and 37 °C treatments with the C3 activation products C3b α′ and iC3b α′-68 is also apparent (Supplementary Fig. S10), providing further evidence that ensilication facilitates the functional retention of Sbi III-IV under conditions of thermal denaturation. Consistent with Fig. 4i, the presence of dimeric Sbi III-IV which has also undergone opsonisation with C3b α′, is visible in all of the released samples.

## Discussion

The cold chain, warranted by the thermal instability of vaccines, remains a major economic and logistical hurdle to global vaccine distribution. Despite technological advances in refrigeration equipment, such as the development of portable solar-powered refrigerators^48,49^, and improvements in monitoring heat exposure through vaccine vial monitors^50^, obstacles at the last mile mean safe and effective vaccines fail to reach the final 15–20% of the unimmunised population^51^. Moreover, the maintenance of a continuous and reliable supply chain at 2–8 °C is likely to become more challenging as the global temperature rises and the climate becomes more unpredictable. Thus, storage and transportation of vaccines at ambient temperatures is a major goal in the field of vaccinology and the development of thermostable vaccines has been named a priority research area in the World Health Organisation’s Global Vaccine Action Plan (2011–2020)^52^.

Silica, a highly-abundant, non-toxic and biocompatible chemical compound formed through hydrolysis and polymerisation of inorganic monomers such as tetraethyl orthosilicate during the sol-gel process, has been shown to enhance protein stability. For example, encapsulation of haemoglobin in transparent mesoporous silica matrices has demonstrated that silica is able to structurally stabilise proteins by sterically hindering conformational changes^53^. In addition, introduction of haemoglobin into the pores of folded-sheet mesoporous silica and the containment of lysozyme, α-lactalbumin and apomyoglobin in silica sheets has been reported to enhance the thermal stability of these proteins^54,55^. More recently, ensilication, a simple yet powerful low-cost method based on the growth of silica cages around proteins, has been shown to confer the model protein HEWL as well as haemoglobin and TTCF with resistance against thermal denaturation^14^.

In this paper we endeavoured to evaluate the use of ensilication as a method for improving the thermal stability of the *M*. *tuberculosis* antigen 85b, the *S*. *aureus* protein-based vaccine adjuvant Sbi III-IV and a novel Sbi III-IV-Ag85b vaccine conjugate. Ag85b is a key component of many TB candidate vaccines in development. These are urgently needed to supplement or replace the current BCG vaccine in order to reduce the number of TB cases and TB-related deaths by prevention, particularly as drug-resistant *M*. *tuberculosis* infections remain high. In this regard, we have recently demonstrated the use of domains III and IV of the *S*. *aureus* immunomodulator Sbi as an adjuvant with the potential of improving the immunogenicity of Ag85b. When co-administered or as a conjugate with Ag85b, Sbi III-IV promotes opsonisation of the antigen with C3 activation products via AP-mediated C3 consumption leading to an enhanced immune response *in vivo* as measured by IgG titre^39^. The thermal stability of Sbi III-IV is relevant to its potential application as an adjuvant in vaccine formulations, particularly as we have shown that Sbi III-IV does not need to be conjugated to Ag85b to enhance its immunogenicity. In addition, both antigen and adjuvant constituents can contribute to the temperature-mediated destabilisation of vaccines and subsequent reductions in efficacy. For example, freeze-thaw cycles of vaccines containing aluminium salt-based adjuvants can cause alum agglomeration and considerably lower potency^56^.

In the first instance we recombinantly expressed and purified our Ag85b, Sbi III-IV-Ag85b and Sbi III-IV constructs (Fig. 1, Supplementary Fig. S1 and Table S1) and subjected them to thermal stability tests. Overall, we found all three constructs showed varying degrees of thermal instability. Ag85b, Sbi III-IV-Ag85b and Sbi III-IV respectively displayed irreversible, partially and fully reversible thermal unfolding with melting temperatures of 53.9 °C, 46.3 °C and 35.6 °C (Figs 2a,b, 3a,b and 4a,b). Although the melting temperature of our Ag85b construct is lower than that previously reported (73.7 °C)^47^, this can be attributed to the small differences in length, molecular weight and secondary structure of the Ag85b constructs used here (Fig. 1a, Supplementary Table S1) and in previous studies^47,57^. All three vaccine-relevant proteins also demonstrated signs of degradation, aggregation and unfolding when exposed to harsh short-term conditions of thermal denaturation (heating at 95 °C for 2.5 hours) or longer-term aging (at 21 °C or 37 °C for up to 7 days) (Figs 2c, 3c and 4c; Supplementary Figs S2–S5, S8 and S9). Furthermore, there was clear evidence of temperature-induced loss of Sbi III-IV-Ag85b and Sbi III-IV functional activity (Figs 3l and 4d).

**Figure 5:**
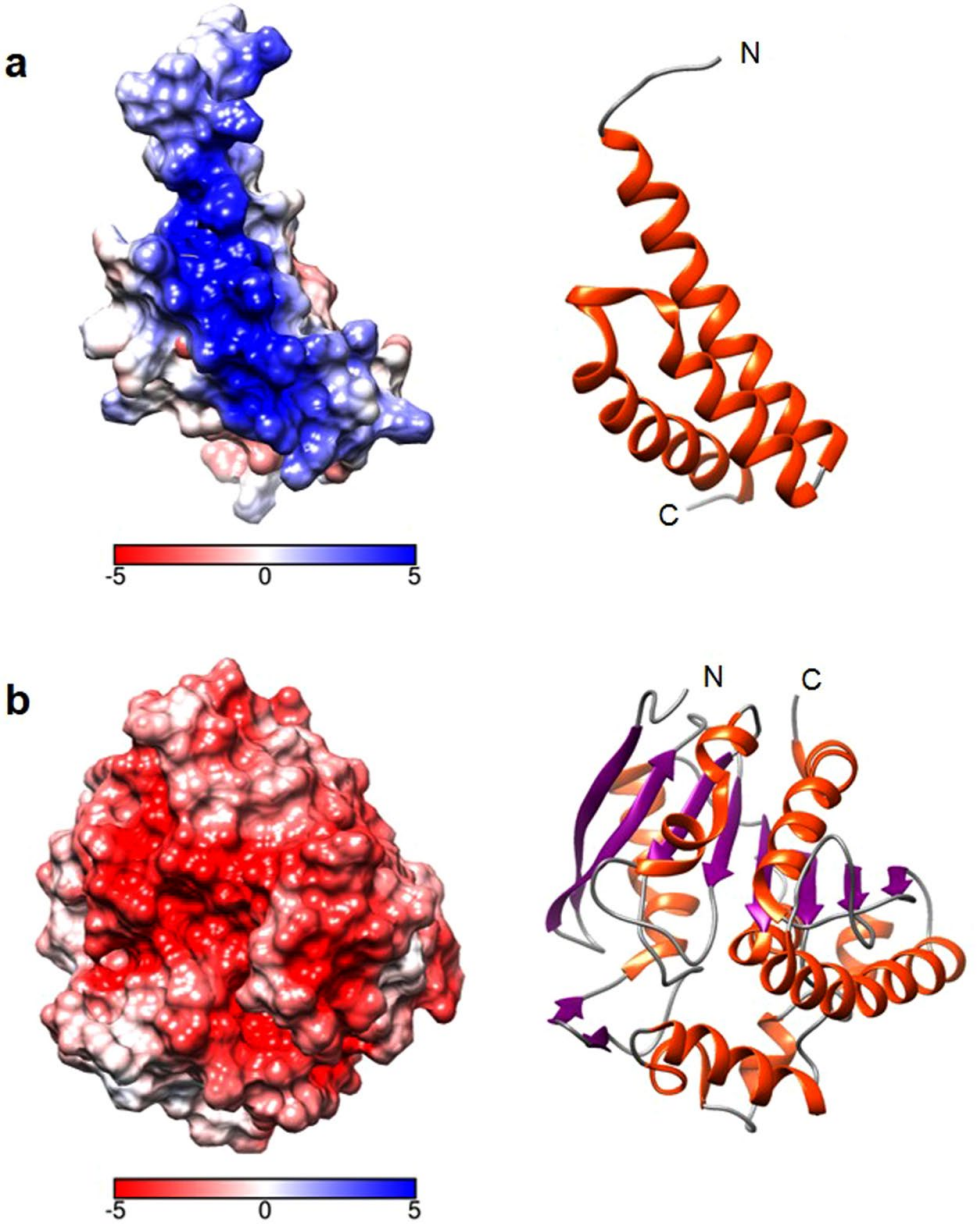
Electrostatic potential surface charge distribution in Sbi IV (**a**) and Ag85b (**b**). The helix α2 surface of Sbi IV is largely positively charged (shown in blue) whereas Ag85b is primarily negatively charged (shown in red). Ribbon diagrams coloured by secondary structure in the same relative positions as the surface models are shown for comparison on the right. All images were generated in UCSF Chimera (version 1.13.1) using the PDB accession codes 2JVH (Sbi IV) and 1F0N (Ag85b).

Now that we had established the thermal instability of Ag85b, Sbi III-IV-Ag85b and Sbi III-IV, we were presented with the opportunity to examine the utility of ensilication in improving the thermal stability of these vaccine-relevant proteins and its application in a vaccine setting for the first time. Ensilication of Sbi III-IV using the standard protocol was successful, producing a powder containing spherical particles of varying sizes (Fig. 4e and Supplementary Fig. S7). This was likely facilitated by the highly positively-charged character of the construct (pI: 9.27) mainly located in the Sbi IV domain (Fig. 5a), enabling electrostatic interactions with silica. However, ensilication of Ag85b and Sbi III-IV-Ag85b in the buffer conditions described by Chen *et al*.^14^ failed to produce ensilicated protein precipitates of a size that was sufficient to yield a solid state material required for thermostabilisation (Figs 2d and 3d,e). This outcome was most likely due to the relatively low pIs of the constructs (Ag85b, pI: 5.65; Sbi III-IV-Ag85b, pI: 6.53) and lack of prominent positively-charged patches on the surface of Ag85b (Fig. 5b). In terms of ensilication, these properties are unfavourable as they may hamper charge-drive interactions between silica and the protein.

Trials designed to promote non-covalent electrostatic interactions between negatively-charged silanol groups and charged amino acid residues showed that although Sbi III-IV, acting as catalyst, improved ensilication of Ag85b and Sbi III-IV-Ag85b, a 50 mM glycine buffer at pH 7.4 was most optimal and was therefore used to produce a solid amorphous ensilicated powder (Figs 2d,e and 3e–h) (Supplementary Tables S2 and S3). From these experiments it was apparent that ensilication using the method described by Chen *et al*.^14^ may be largely limited to positively-charged proteins. We found glycine, a simple amino acid existing as a zwitterion at neutral pH, is able to facilitate ensilication of Ag85b and Sbi III-IV-Ag85b, which are negatively-charged/neutral. Glycine-modified silica nanoparticles, with particle sizes correlating with the amount of glycine, have been produced previously by Mansa and colleagues^58^ using a modified sol-gel method. The authors suggest that the silanol groups (Si-OH) of the hydrolysed tetraethyl orthosilicate silica precursor react with the hydroxyl of the glycine carboxyl group resulting in the formation of covalent silicon esters (Si–O–CO). These esters subsequently undergo polymerisation resulting in the formation of a silica network. It is possible that inclusion of glycine within the sol-gel reaction in our study results in the formation of these glycine-silica interactions which allow ensilication of Ag85b and Sbi III-IV-Ag85b to proceed.

The secondary structures of Ag85b, Sbi III-IV-Ag85b and Sbi III-IV chemically released from the ensilicated material were found to be comparable to their native unensilicated counterparts (Figs 2f, 3i and 4f). Released Sbi III-IV-Ag85b and Sbi III-IV also retained full functionality in terms of their ability to induce AP activation-mediated C3 consumption (Figs 3g,h,j and 4g,h). More importantly, ensilication was able to protect all three proteins against temperature-induced degradation, aggregation (Figs 2g, 3k and 4i; Supplementary Figs S2, S4 and S8) and unfolding (Supplementary Figs S3, S5 and S9). Ensilication also prevented the loss of functional activity of Sbi III-IV-Ag85b and Sbi III-IV caused by thermal denaturation, which was clearly affected in their native equivalents (Figs 3l and 4j; Supplementary Figs S6 and S10). We have thus demonstrated the potential benefits of ensilication in the storage and transport of Ag85b-containing vaccines at ambient temperatures and provided support for this method in thermostabilising adjuvants such as Sbi III-IV in the future. Interestingly, the formation of denaturant-resistant homodimers was visible in all of the released Sbi III-IV samples (Fig. 4i and Supplementary Fig. S10), suggesting the ensilication and/or release process promotes self-association of this protein which has a tendency to dimerise in the absence of ensilication as well. However, the amount of dimer compared to monomer in these samples was relatively small and did not appear to have any deleterious effects on protein function as their ability to mediate C3 depletion and C3 activation-mediated opsonisation was comparable to native untreated Sbi III-IV (Fig. 4j and Supplementary Fig. S10).

Although the results of the *in vitro* experiments presented here are encouraging, in the future the functionality of released Sbi III-IV and Sbi III-IV-Ag85b should also be evaluated *in vivo*. In addition, the functionality of released Ag85b should be compared to the native antigen, although given the results obtained with the proteins evaluated here and by Chen and colleagues^14^, there is little reason to suggest that ensilication would have any detrimental effects on the function of the antigen. Furthermore, although the method employed here and previously^14^ provides further proof of concept that the dissolution of silica by acidic fluoride solutions is a reliable^59^ and efficient release protocol, development of a more practical method of releasing proteins from ensilicated material, suitable for use in the field, is  desirable.

In summary, here we provide an in-depth analysis of the longer-term thermal instability of Ag85b, a key player in the development of new TB vaccines. We have also followed-up on Yang *et al*.’s^39^ paper and further characterised the *S*. *aureus*-based vaccine adjuvant Sbi III-IV and the Sbi III-IV-Ag85b conjugate by revealing their sensitivity to thermal denaturation. Moreover, we provide evidence that ensilication, a method based on growth of a silica matrix around proteins, is capable of stabilising Ag85b, the Sbi III-IV-Ag85b conjugate and the Sbi III-IV adjuvant against temperature-induced loss of structure and function without the need for refrigeration or lyophilisation. In so doing we have overcome the limitation of ensilication being restricted to positively-charged proteins by identifying conditions that can be used to ensilicate neutral or negatively-charged proteins and have thus broadened the application of this technique. Overall, the results presented could have widespread implications for the thermostabilisation of vaccine-relevant proteins and hence vaccine distribution in the future.

## Materials and Methods

### Protein constructs

The antigen 85b protein construct was comprised of 285 amino acids corresponding to the Ag85b sequence while the Sbi III-IV-Ag85b conjugate designed previously contained Sbi domains III and IV conjugated via a 28 amino acid histidine and glycine-rich linker to Ag85b at the C-terminus^39^. The Sbi III-IV construct created previously^60^ was composed of the amino acid sequence corresponding to Sbi domains III and IV (153–253 of full-length Sbi). All three recombinant proteins bore an N-terminal 6x His-tag which in the Ag85b and Sbi III-IV-Ag85b constructs was followed by a thrombin cleavage site.

### Expression of recombinant Ag85b, Sbi III-IV-Ag85b and Sbi III-IV

All three protein constructs were expressed in the *Escherichia coli* BL21(DE3) expression strain. Briefly, *E*. *coli* BL21 cells harbouring the pOPINF-Ag85b or pET15b-Sbi III-IV-Ag85b plasmids were grown at 37 °C in Luria-Bertani (LB) broth supplemented with 100 μg ml^−1^ ampicillin until an OD_600_ of 0.4–0.6. Protein expression was then induced with 500 μM Isopropyl β-D-1-thiogalactopyranoside (IPTG) at 16 °C for 16 hours. For cells bearing the pQE30-Sbi III-IV plasmid, protein expression was induced at 37 °C for 3 hours after an OD_600_ of 0.6–0.8 was reached. Harvested cells were lysed by sonication in the presence of a protease inhibitor cocktail (set VII, Merck) and the resultant lysate was clarified by centrifugation.

### Protein purification

Primary purification was performed using nickel-affinity chromatography. His-tagged Ag85b, Sbi III-IV-Ag85b and Sbi III-IV proteins were eluted from a 1 ml pre-packed HisTrap column (GE Healthcare) using a linear gradient of 0.0–0.5 M imidazole in 50 mM Tris, 150 mM NaCl pH 7.4. Fractions containing the desired protein were pooled and exchanged into a buffer suitable for ion exchange chromatography (Ag85b: 20 mM Bis Tris pH 6.6, Sbi III-IV-Ag85b: 20 mM Tris pH 7.6, Sbi III-IV: 50 mM HEPES pH 8). Ag85b and Sbi III-IV-Ag85b were subjected to anion exchange (HiTrap Q, GE Healthcare) while Sbi III-IV was cation-exchanged (HiTrap SP, GE Healthcare). Proteins were eluted from the columns with a linear gradient of NaCl (0.0–1.0 M). In order to further improve purity, Ag85b, Sbi III-IV-Ag85b and Sbi III-IV were subsequently purified using size exclusion chromatography with a HiLoad 16/600 Superdex S200 column (GE Healthcare) equilibrated in 20 mM Tris, 150 mM NaCl pH 7.4. Fractions containing the most pure protein were concentrated and protein concentration measured using a BCA assay (Thermo Scientific).

### Circular dichroism (CD) spectroscopy

Buffer-corrected CD measurements of 50 μg ml^−1^ thermally-treated native and released Ag85b, Sbi III-IV-Ag85b and Sbi III-IV in 10 mM sodium phosphate pH 7.4 were acquired at 20 °C. For native untreated samples only, measurements were obtained at temperatures ranging from 5 °C to 85 °C in 5 °C increments followed by a final reading at 20 °C. All measurements were obtained on a Chirascan spectrometer (Applied Photophysics) at wavelengths between 190 and 280 nm in 1 nm increments with a bandwidth of 2 nm and 2 second time per point. Millidegree values were converted to delta epsilon units and deconvolution was performed using DichroWeb. Melting temperature values were estimated using sigmoidal fits of thermal denaturation curves at 222 nm performed using the OriginLab (version 2018b) Boltzmann function.

### Ensilication

Ensilication of Ag85b, Sbi III-IV-Ag85b and Sbi III-IV was performed using modifications of the protocol described previously^14^. Briefly, tetraethyl orthosilicate (TEOS) (Sigma Aldrich) was hydrolysed and mixed with 1 mg ml^−1^ of protein in 50 mM Tris pH 7 at a ratio of 1:50 to enable condensation and polymerisation as per the standard protocol. Other conditions trialled included 50 mM Tris pH 7 with the addition of 20 mM calcium chloride, 20 mM magnesium chloride, 20 mM lysine, 20 mM sodium chloride or 1% polyethylene glycol 400, 50 mM glycine pH 7.4, 10 mM sodium phosphate pH 7.4 or increasing concentrations (0.02–0.3 mg ml^−1^) of Sbi III-IV. In the 96-well plate format, plates were examined for signs of visible precipitate formation after 2 hours. For small-scale (1 ml) trials, ensilication was allowed to proceed for 1 hour after which the material was centrifuged at 14,500 × *g* and the unensilicated supernatant retained. The pellet was subsequently washed with 200 µl MilliQ water, centrifuged a second time and the pellet allowed to dry for 24–48 h to enable the production of a dry powder. Concentration of the protein within the unensilicated supernatant and ensilication wash was measured using a BCA assay (Thermo Scientific).

For ensilication on a larger scale (>4 ml), the ensilicated material and subsequent wash were filtered through a glass microfiber filter with 0.7 μm retention (Thermo Scientific) using a Buchner vacuum filtration system. Ensilicated particles retained on the filter were left to dry in an extractor for 24–48 h and the resultant ensilicated powder was subsequently collected and weighed. The filtration flowthrough and ensilication wash were analysed for the presence of unensilicated protein using a BCA assay (Thermo Scientific).

### Chemical release of ensilicated material

Release of ensilicated proteins back into solution was performed using the protocol detailed by Chen *et al*.^14^ Briefly, 0.5 ml of 50 mM Tris pH 7 and 190 mM sodium fluoride pH 2.9 was added to 1 mg of ensilicated powder. Following rotation at room temperature for 1 h, silica particulates were no longer visible and the protein samples were buffer-exchanged into 20 mM Tris, 150 mM NaCl pH 7.4 for further analysis.

### Dynamic light scattering (DLS)

To analyse the increase in size of ensilicated Sbi III-IV-Ag85b particles over time, prehydrolysed TEOS was added to 100 µl 1 mg ml^−1^ Sbi III-IV-Ag85b in 50 mM Tris pH 7 at a ratio of 1:50. The volume of the solution was subsequently adjusted to 1 ml in order to retard the ensilication process and capture intermediate species. Particle size was subsequently monitored for 30 minutes with measurements acquired in intervals of 1 minute. To compare the sizes of untreated and thermally-treated native and released Ag85b, Sbi III-IV-Ag85b and Sbi III-IV, 50 µl samples at a concentration of 0.2 mg ml^−1^ were used. All particle size measurements according to volume and intensity were performed on a Zetasizer Nano S dynamic light scattering (DLS) instrument (Malvern) using disposable plastic micro cuvettes (ZEN0040, Malvern).

### Field emission scanning electron microscopy (FESEM)

Ensilicated Sbi III-IV powder particles were fixed to carbon-coated tape and desiccated overnight. Particles were subsequently viewed under a scanning electron microscope (6301 F, JEOL Ltd.) (Centre for Electron Optical Studies, University of Bath) and images at different magnifications (650–30,000×) were captured.

### Temperature treatment

Native solutions or samples of Ag85b, Sbi III-IV-Ag85b and Sbi III-IV ensilicated powder were boiled at 95 °C for 2.5 hours or flash-frozen in liquid nitrogen and thawed at room temperature three times in order to probe heating and freezing stability. Longer-term stability was analysed by incubation at 4 °C, room temperature (21 °C) or 37 °C for up to 7 days. Ensilicated Sbi III-IV was also aged at room temperature for 7 months. Following the temperature treatments, ensilicated samples were released and buffer-exchanged as described above. A cocktail of protease inhibitors (set VII, Merck) was added to all native protein solutions to reduce the appearance of effects caused by any contaminating proteases. Following treatment, the protease inhibitors were removed by buffer-exchange. Degradation and aggregation of native and released samples was visualised using DLS and SDS-PAGE with 15% resolving gels and temperature-induced unfolding was analysed using CD.

### Western blot analysis of C3 consumption

10 μl of pooled complement human serum (Patricell) was mixed with an equal volume of untreated or thermally-treated native or released Sbi III-IV-Ag85b or Sbi III-IV. Following incubation at 37 °C for 1 h, 1 μl of the serum solution was loaded onto an 8% resolving SDS-PAGE gel and electrophoresed under reducing conditions. Proteins were transferred onto a PVDF membrane, probed with a rabbit polyclonal anti-Sbi primary antibody (1.5:5,000 dilution, kind gift from Professor Tim Foster, Trinity College Dublin) and detected using a goat anti-rabbit HRP-conjugated secondary antibody (1:2,500 dilution, Thermo Fisher).

### Analysis of alternative pathway (AP) activity

AP activity was analysed using the ELISA-based Wieslab complement system AP screen (Euro Diagnostica). Samples were prepared by mixing 10 μl of native or released Sbi III-IV-Ag85b (4 μM) or Sbi III-IV (2 μM) with an equal volume of normal human serum (NHS) (Euro Diagnostica). Following incubation at 37 °C for 30 min, the treated serum samples were diluted 1:16 with AP diluent, blocking activation of the classical and lectin pathways. Diluted serum samples were loaded onto lipopolysaccharide-coated wells in duplicate along with a blank (AP diluent), positive (NHS) and negative (heat-inactivated NHS) control. From this point the manufacturer’s instructions were followed. Blank-corrected absorbance measurements at 405 nm correlating with the level of complement activation were obtained and converted to residual AP activity (%) using Equation 1.

1 $${\rm{Residual}}\,{\rm{AP}}\,{\rm{activity}}\,( \% )=\frac{{\rm{Sample}}-{\rm{negative}}\,{\rm{control}}}{{\rm{Positive}}\,{\rm{control}}-{\rm{negative}}\,{\rm{control}}}\times {\rm{100}}$$

Data were expressed as mean values from at least 2 replicates ± standard deviation from the mean and depicted as bar charts using GraphPad Prism (version 7.04). As Sbi III-IV causes AP activation and depletes serum C3 levels, functional Sbi III-IV or Sbi III-IV-Ag85b produces a result similar to the negative control (heat-inactivated serum).

### Statistical analysis

Statistically significant differences in AP activity were determined using GraphPad Prism (version 7.04). A one-way ANOVA with Dunnett’s multiple comparisons analysis was used unless only two datasets were being compared in which case an unpaired t-test was performed. *P* values < 0.01 were considered statistically significant.

## Supplementary information

Supplementary Information

## Data Availability

The authors will willingly grant requests made by qualified researchers for the raw data generated or analysed in this published article.
