# Supplementary material for: Ensilication Improves the Thermal Stability of the Tuberculosis Antigen Ag85b and an Sbi-Ag85b Vaccine Conjugate
Source: Sci Rep. 2019 Aug 8;9:11409. doi: 10.1038/s41598-019-47657-9 (PMC6685958; doi:10.1038/s41598-019-47657-9)
Supplement: Supplementary file 1 — Supplementary Information [file 41598_2019_47657_MOESM1_ESM.pdf]

Supplementary Information for:

Ensilication Improves the Thermal Stability of the Tuberculosis Antigen Ag85b and an Sbi-Ag85b Vaccine Conjugate

Wahid, AA.[1], Doekhie, A.[2], Sartbaeva, A.[2] and van den Elsen, JMH.[1]

[1]Department of Biology and Biochemistry, University of Bath, Bath, UK

[2]Department of Chemistry, University of Bath, Bath, UK

Address correspondence to: Jean van den Elsen (J.M.H.V.Elsen@bath.ac.uk)
or Asel Sartbaeva (A.Sartbaeva@bath.ac.uk)

This PDF file includes:

Supplementary Figures S1 – S22

Supplementary Tables S1 – S3

Contents of Supplementary Information, Wahid *et al*.,:

Ensilication Improves the Thermal Stability of the Tuberculosis Antigen Ag85b and an Sbi-Ag85b Vaccine Conjugate

Supplementary Figures

**Supplementary Figure S1.**	**Chromatograms showing elution peaks of Ag85b (**top**), Sbi III-IV-Ag85b (**centre**) and Sbi III-IV (**bottom**) nickel affinity (**left**), ion exchange (**centre**) and size exclusion (**right**) chromatography.**
**Supplementary Figure S2.**	**Particle size analysis of thermally-treated native (left) and released (right) Ag85b.**
**Supplementary Figure S3.**	**Secondary structure analysis of thermally-treated native and released Ag85b.**
**Supplementary Figure S4.**	**Particle size analysis of thermally-treated native (left) and released (right) Sbi III-IV-Ag85b.**
**Supplementary Figure S5.**	**Secondary structure analysis of thermally-treated native and released Sbi III-IV-Ag85b**
**Supplementary Figure S6.**	**Analysis of opsonisation following C3 activation by thermally-treated released Sbi III-IV-Ag85b.**
**Supplementary Figure S7.**	**FESEM images of ensilicated Sbi III-IV powder at 650x (left) and 5,000x (right) magnification.**
**Supplementary Figure S8.**	**Particle size analysis of thermally-treated native (left) and released (right) Sbi III-IV.**
**Supplementary Figure S9.**	**Secondary structure analysis of thermally-treated native and released Sbi III-IV.**
**Supplementary Figure S10.**	**Analysis of opsonisation following C3 activation by thermally-treated released Sbi III-IV.**
**Supplementary Figures S11-S22.**	**Uncropped SDS-PAGE gel and western blot images**

Supplementary Tables

**Supplementary Table S1.**	**Physiochemical properties of the Ag85b, Sbi III-IV-Ag85b and Sbi III-IV protein constructs.**
**Supplementary Table S2.**	**Concentrations of protein present in the released solution, unensilicated supernatant and ensilication wash following Ag85b ensilication under two different conditions.**
**Supplementary Table S3.**	**Concentrations of protein present in the released solution, unensilicated supernatant and ensilication wash following Sbi III-IV-Ag85b ensilication under four different conditions.**

Supplementary Figure S1.

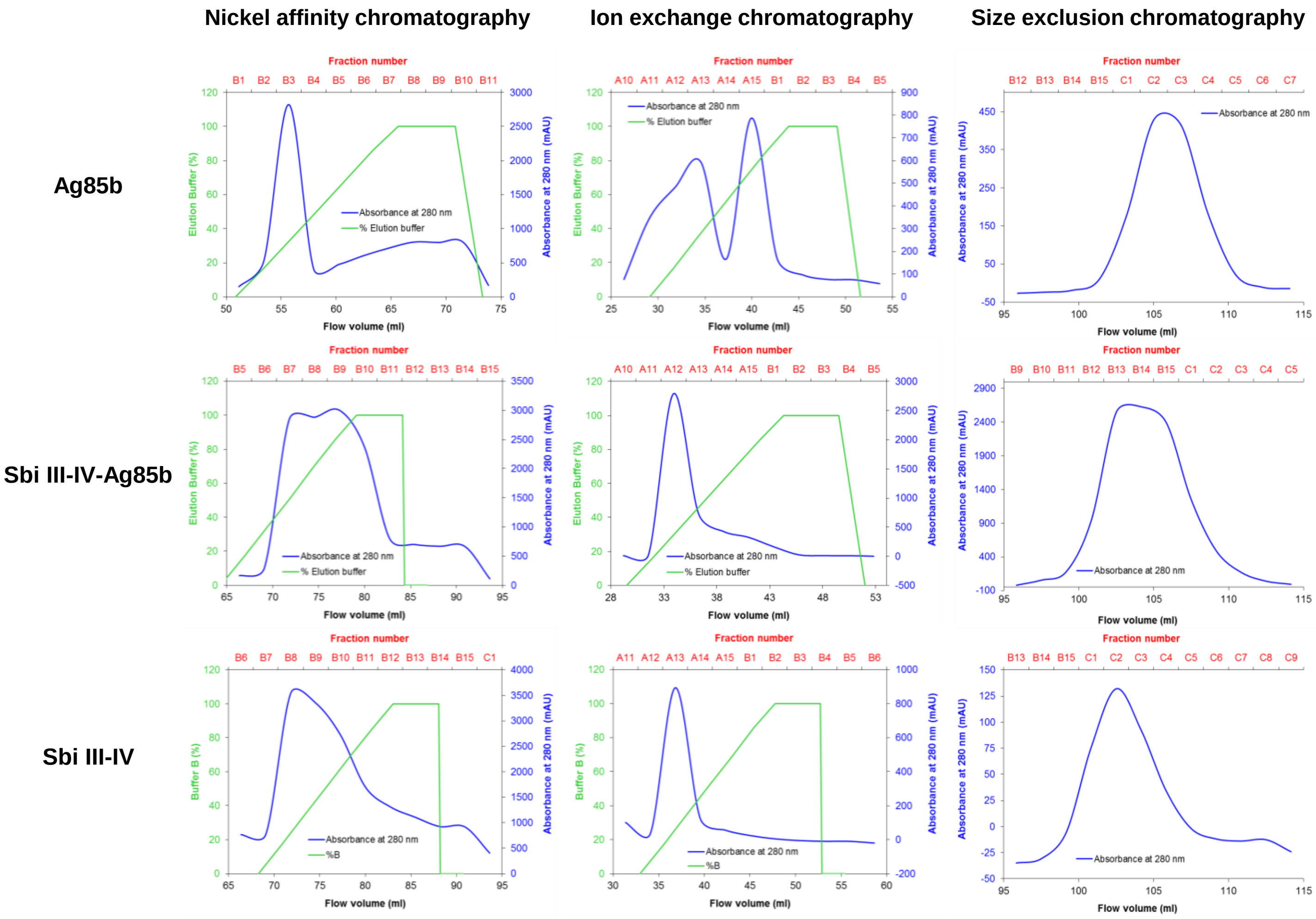

Supplementary Figure S1. Chromatograms showing elution peaks of Ag85b (top)**, Sbi III-IV-Ag85b** (middle) **and Sbi III-IV** (bottom) **nickel affinity** (left)**, ion exchange** (middle) **and size exclusion** (right) **chromatography.**

Supplementary Figure S2.

Native | **Released**

Untreated

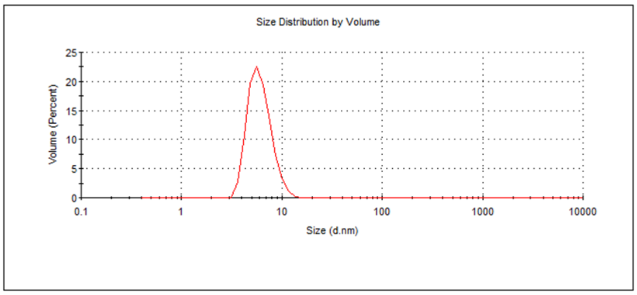

Size (d.nm):	Volume (%):	St Dev (d.nm):	PdI
6.139	100.0	1.648	0.258

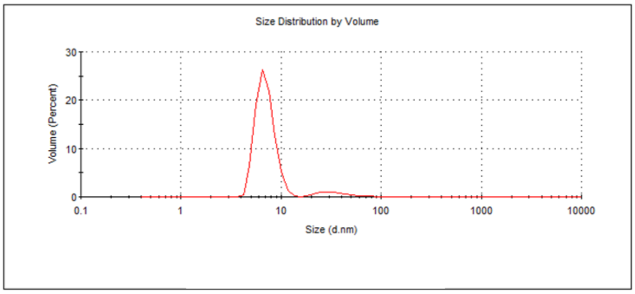

Size (d.nm):	Volume (%):	St Dev (d.nm):	PdI
7.033	93.4	1.504	0.416

95 °C 2.5 h

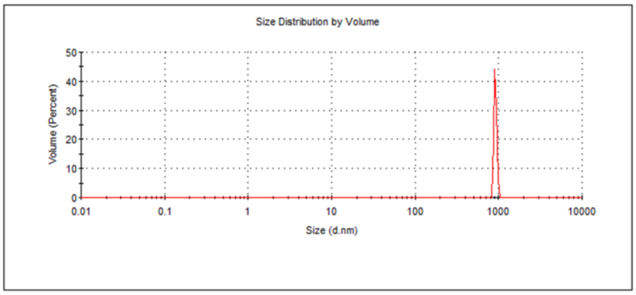

Size (d.nm):	Volume (%):	St Dev (d.nm):	PdI
915.4	100.0	35.04	0.519

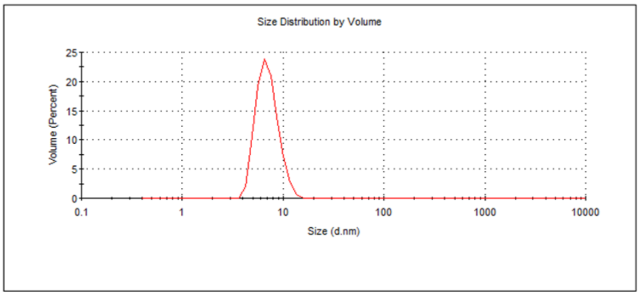

Size (d.nm):	Volume (%):	St Dev (d.nm):	PdI
7.112	100.0	1.764	0.537

37 °C 7 days

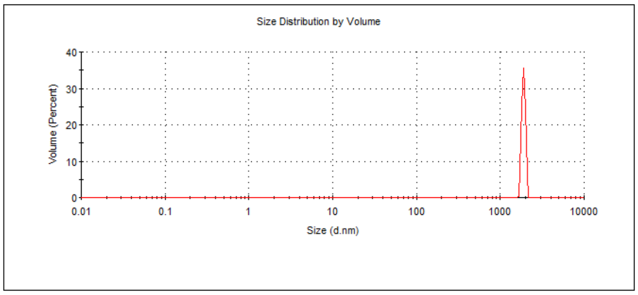

Size (d.nm):	Volume (%):	St Dev (d.nm):	PdI
1894	100.0	91.44	0.497

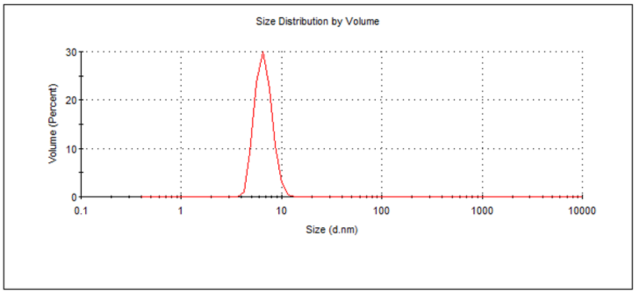

Size (d.nm):	Volume (%):	St Dev (d.nm):	PdI
6.704	99.9	1.290	0.502

Supplementary Figure S2. Particle size analysis of thermally-treated native (left**) and released (**right**) Ag85b.** Dynamic light scattering size distribution by volume plots showing comparable sizes of untreated native, and ensilicated and subsequently released Ag85b (top). Large aggregates occur in the native but not released Ag85b samples following treatment at 95°C for 2.5 h (middle) or 37°C for 7 days (bottom). Plots are representative of ≥11 measurements. PdI: polydispersity index.

Supplementary Figure S3.

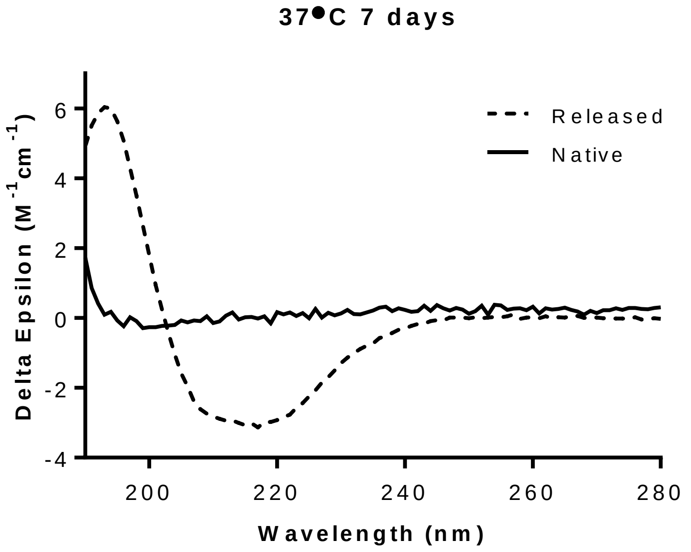

	Reference set for CDSSTR	α helices	β sheets	Turns	Unordered	Total	NRMSD
**Native**	4	7%	36%	15%	39%	97%	0.134
**Released**	4	17%	31%	21%	30%	99%	0.011

Supplementary Figure S3. Secondary structure analysis of thermally-treated native and released Ag85b. CD spectra showing ensilicated Ag85b released following 7 days at 37°C remains folded with a similar secondary structure to native untreated Ag85b (shown in Figure 2f). On the other hand, native Ag85b shows substantial unfolding when subjected to the same treatment. Displayed spectra are representative of 3 replicates. Deconvolution was performed using DichroWeb.

Supplementary Figure S4.

Native | **Released**

Untreated

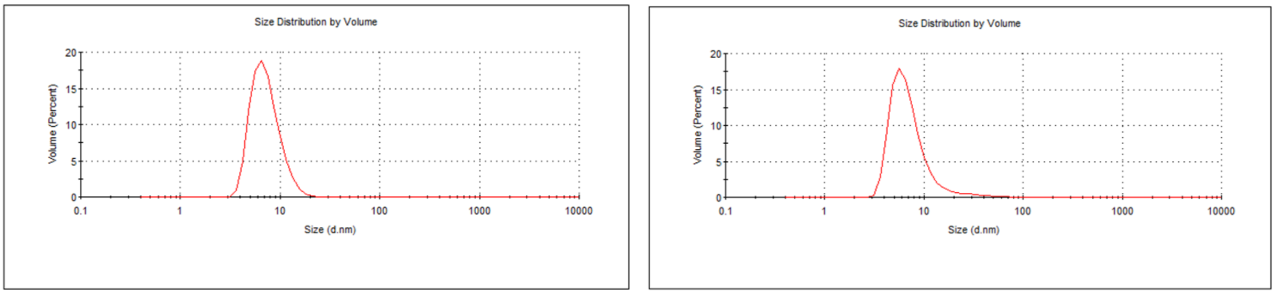

Size (d.nm):	Volume (%):	St Dev (d.nm):	PdI	Size (d.nm):	Volume (%):	St Dev (d.nm):	PdI
7.329	100.0	2.457	0.231	7.723	99.8	6.412	0.723

95 °C 2.5 h

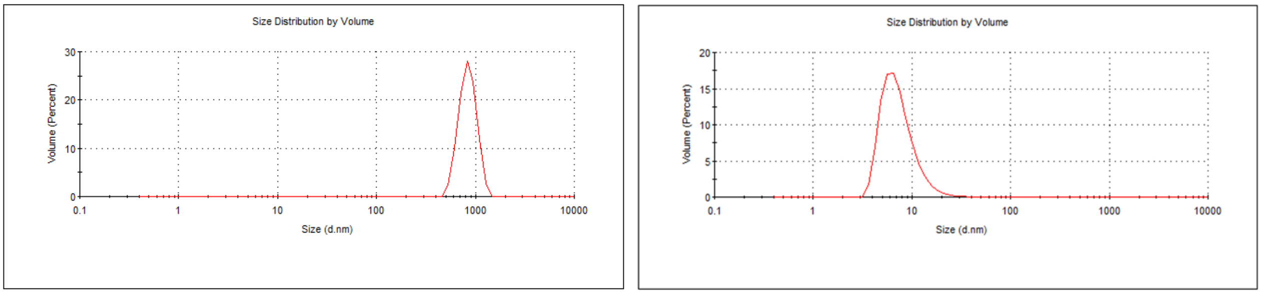

Size (d.nm):	Volume (%):	St Dev (d.nm):	PdI	Size (d.nm):	Volume (%):	St Dev (d.nm):	PdI
849.8	100.0	166.1	0.422	7.473	99.9	3.336	0.332

37 °C 7 days

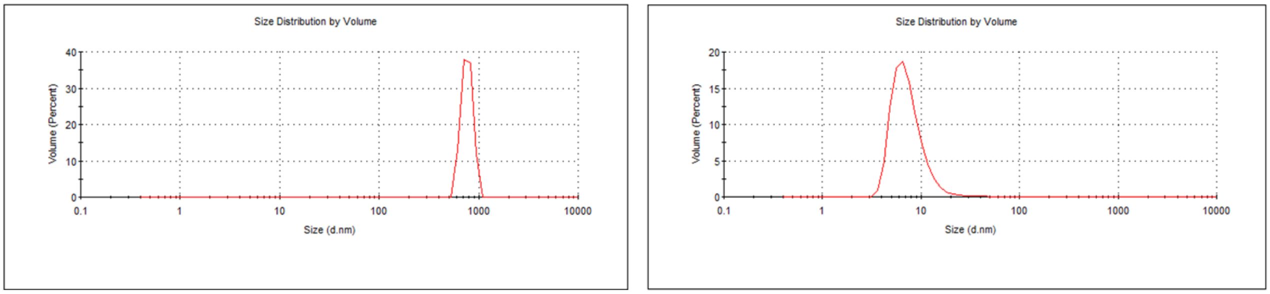

Size (d.nm):	Volume (%):	St Dev (d.nm):	PdI	Size (d.nm):	Volume (%):	St Dev (d.nm):	PdI
770.6	100.0	98.10	0.574	7.659	100.0	4.377	0.141

Supplementary Figure S4. Particle size analysis of thermally-treated native (left**) and released (**right**) Sbi III-IV-Ag85b.** Dynamic light scattering size distribution by volume plots showing comparable sizes of untreated native, and ensilicated and subsequently released Sbi III-IV-Ag85b (top). Aggregate formation and an increase in polydispersity is seen in the native but not released Sbi III-IV-Ag85b samples following treatment at 95°C for 2.5 h (middle) or 37°C for 7 days (bottom). Plots are representative of ≥11 measurements. PdI: polydispersity index.

Supplementary Figure S5.

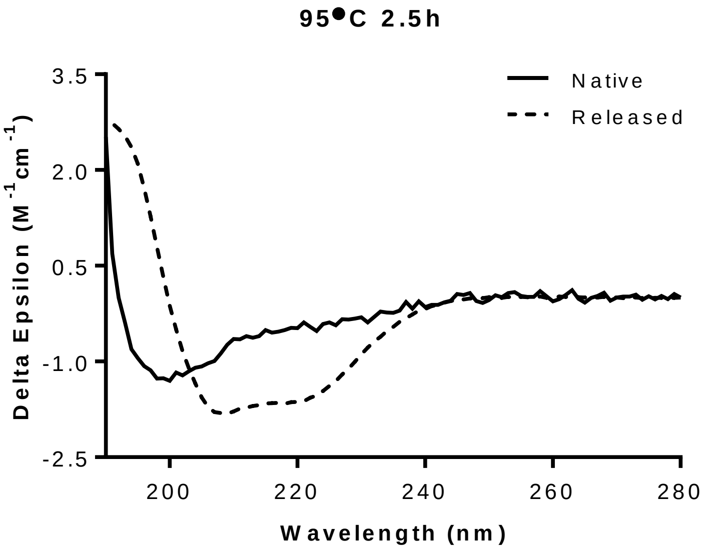

	Reference set for CDSSTR	α helices	β sheets	Turns	Unordered	Total	NRMSD
**Native**	4	6%	35%	25%	33%	99%	0.066
**Released**	4	12%	32%	23%	32%	99%	0.016

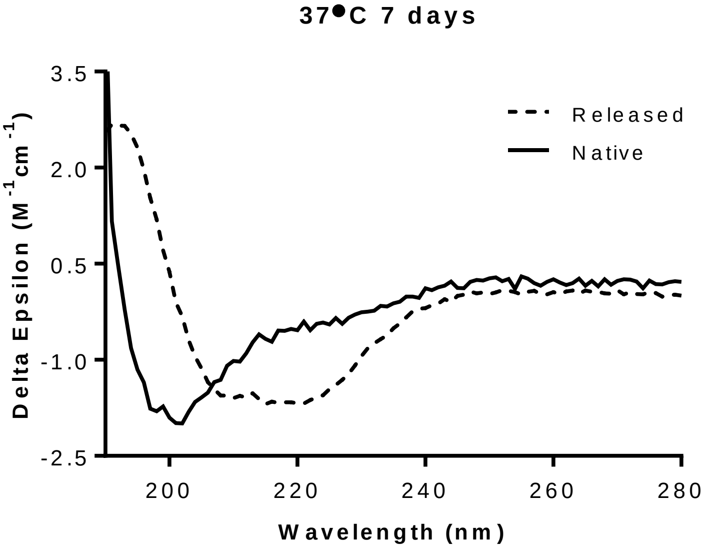

	Reference set for CDSSTR	α helices	β sheets	Turns	Unordered	Total	NRMSD
**Native**	4	5%	35%	24%	34%	98%	0.053
**Released**	4	12%	33%	24%	31%	100%	0.024

Supplementary Figure S5. Secondary structure analysis of thermally-treated native and released Sbi III-IV-Ag85b. CD spectra showing ensilicated Sbi III-IV-Ag85b released following 2.5h at 95°C (top) or 7 days at 37°C (bottom) remains folded with a similar secondary structure to native untreated Sbi III-IV-Ag85b (shown in Figure 3i). On the other hand, native Sbi III-IV-Ag85b shows signs of unfolding and loss of helicity when subjected to the same treatments. Displayed spectra are representative of 3 replicates. Deconvolution was performed using DichroWeb.

Supplementary Figure S6.

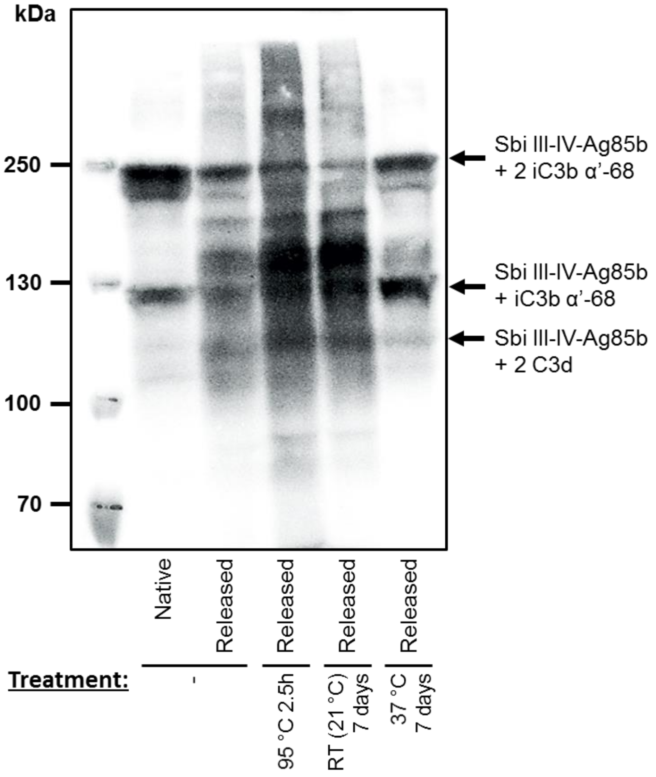

Supplementary Figure S6. Analysis of opsonisation following C3 activation by thermally-treated released Sbi III-IV-Ag85b. Anti-Sbi western blot analysis following a 1 h incubation of normal human serum with 24 µM untreated native or released Sbi III-IV-Ag85b or ensilicated Sbi III-IV-Ag85b released following the thermal treatments shown. Opsonisation of Sbi III-IV-Ag85b with C3 activation products iC3b α'-68 and C3d is evident in all thermally-treated released samples, consistent with the functional activity data shown in Figure 3l and providing further evidence that ensilication facilitates the functional retention of Sbi III-IV-Ag85b under conditions of thermal denaturation.

Supplementary Figure S7.

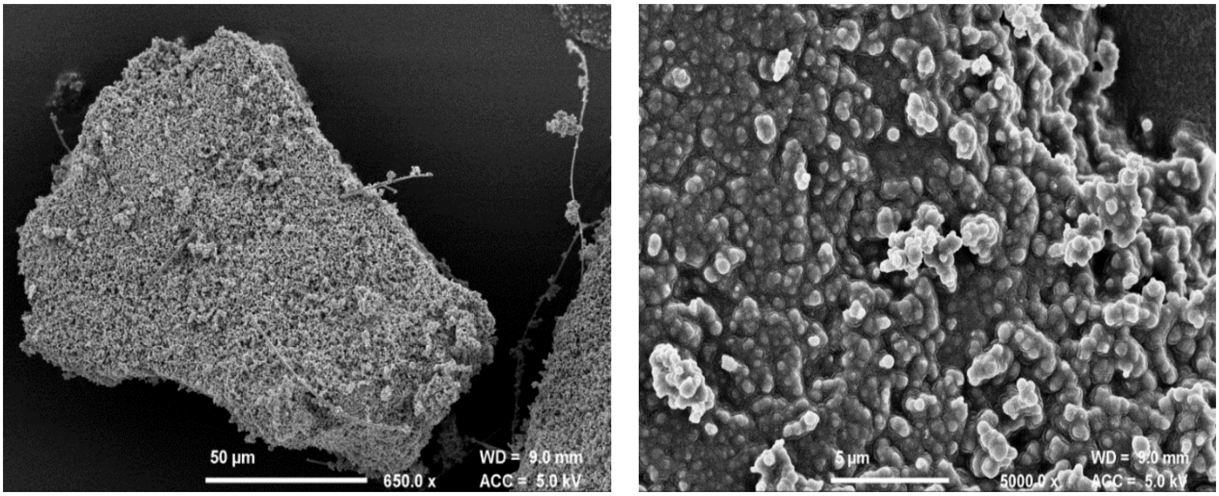

Supplementary Figure S7. FESEM images of ensilicated Sbi III-IV powder at 650x (left**) and 5,000x (**right**) magnification.** Scale bars are shown at the bottom of the images.

Supplementary Figure S8.

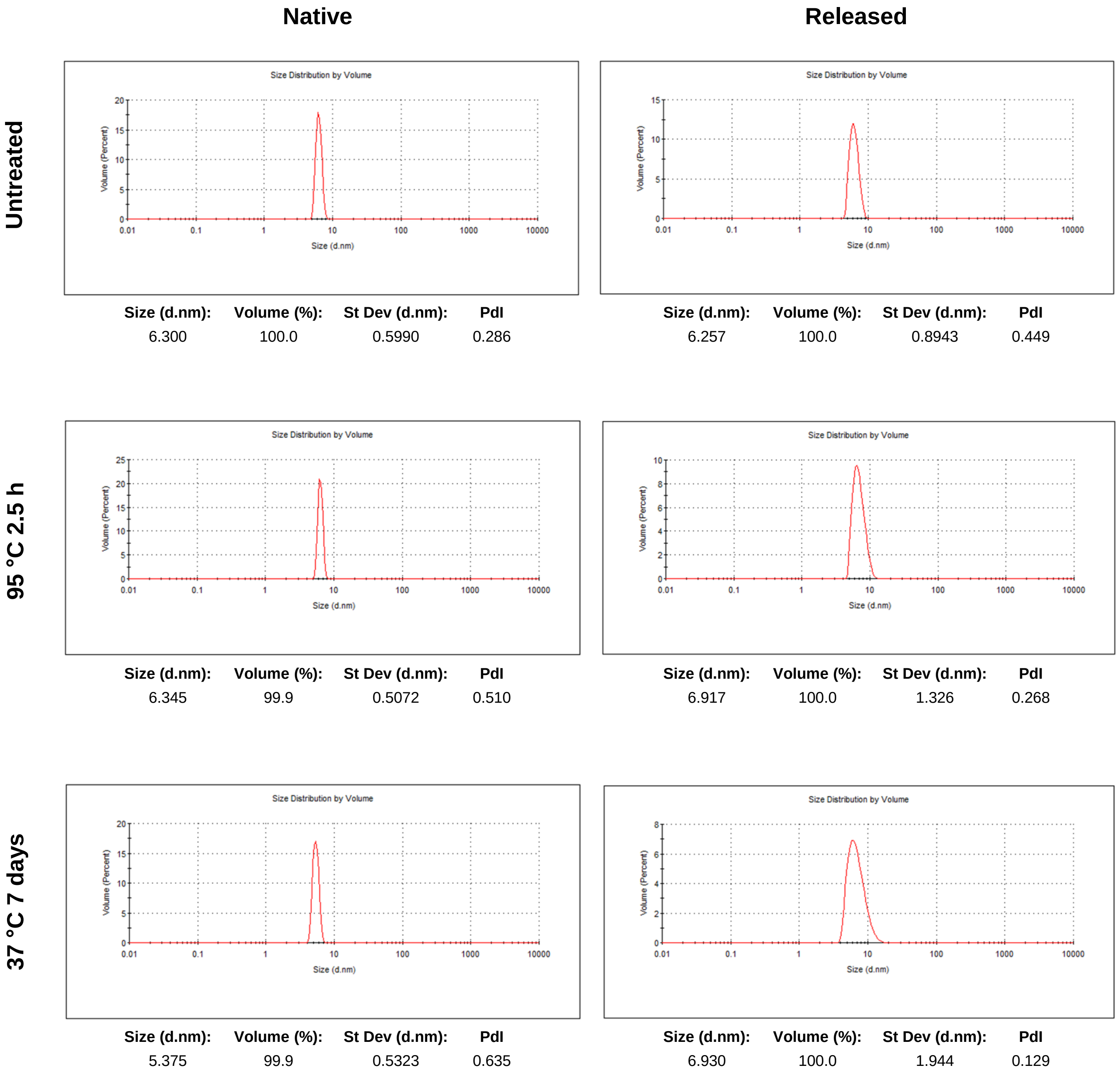

Supplementary Figure S8. Particle size analysis of thermally-treated native (left**) and released (**right**) Sbi III-IV.** Dynamic light scattering size distribution by volume plots showing no substantial differences in the size of native or ensilicated and subsequently released Sbi III-IV with no treatment (top) or following treatment at 95°C for 2.5 h (middle) or 37°C for 7 days (bottom). However, an increase in polydispersity of native Sbi III-IV following the temperature treatments is evident. Plots are representative of ≥11 measurements. PdI: polydispersity index.

Supplementary Figure S9.

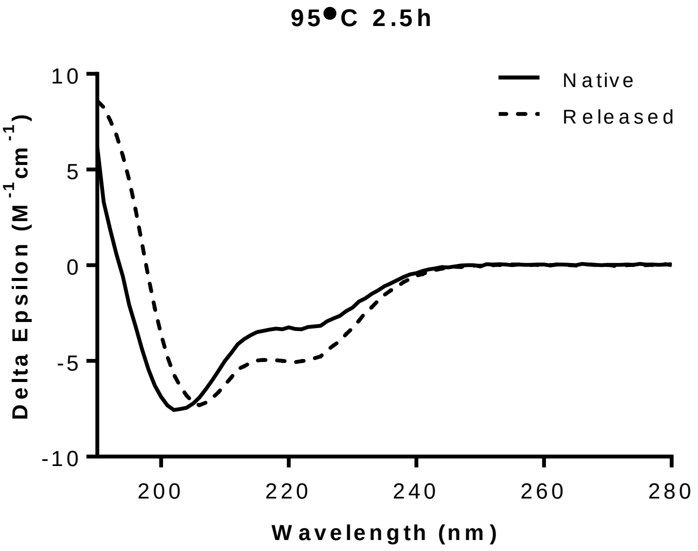

	Reference set for CDSSTR	α helices	β sheets	Turns	Unordered	Total	NRMSD
**Native**	7	24%	16%	17%	43%	100%	0.008
**Released**	7	51%	10%	10%	28%	99%	0.009

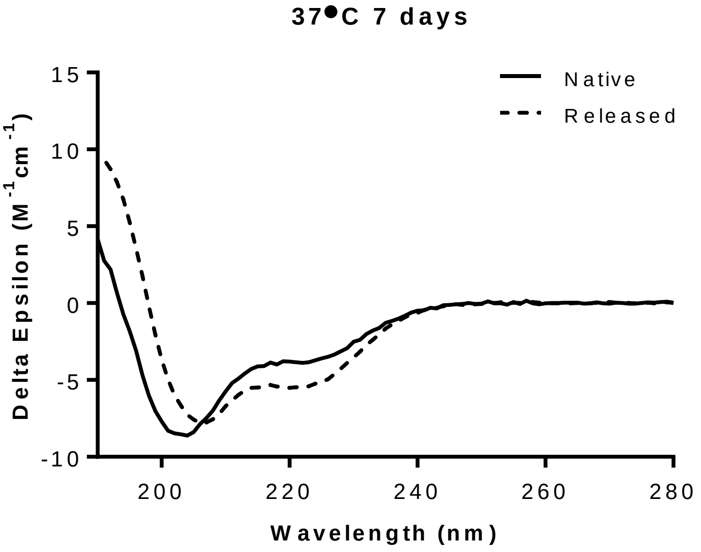

	Reference set for CDSSTR	α helices	β sheets	Turns	Unordered	Total	NRMSD
**Native**	7	39%	15%	13%	33%	100%	0.005
**Released**	7	55%	9%	7%	28%	99%	0.007

Supplementary Figure S9. Secondary structure analysis of thermally-treated native and released Sbi III-IV. CD spectra showing ensilicated Sbi III-IV released following 2.5h at 95°C (top) or 7 days at 37°C (bottom) remains folded with a similar secondary structure to native untreated Sbi III-IV (shown in Figure 4f). On the other hand, native Sbi III-IV shows signs of unfolding and loss of helicity when subjected to the same treatments. Displayed spectra are representative of 3 replicates. Deconvolution was performed using DichroWeb.

Supplementary Figure S10.

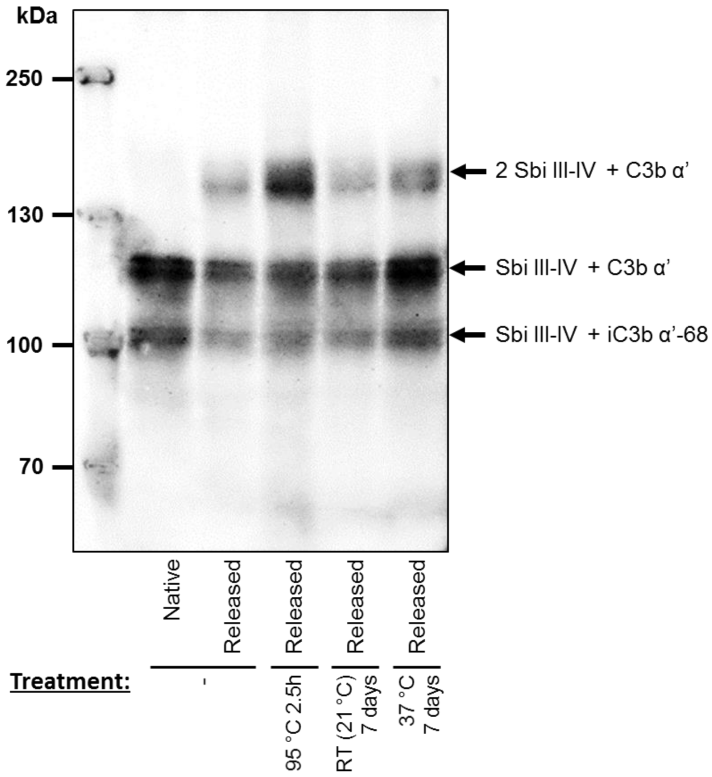

Supplementary Figure S10. Analysis of opsonisation following C3 activation by thermally-treated released Sbi III-IV. Anti-Sbi western blot analysis following a 1 h incubation of normal human serum with 24 µM untreated native or released Sbi III-IV or ensilicated Sbi III-IV released following the thermal treatments shown. Opsonisation of Sbi III-IV with C3 activation products C3b α' and iC3b α'-68 is evident in all thermally-treated released samples. Consistent with Figure 4i, the presence of dimeric Sbi III-IV which has also undergone opsonisation with C3b α' is visible in all of the released samples. Further to the functional activity data presented in Figure 4j, these results provide further evidence that ensilication facilitates the functional retention of Sbi III-IV under conditions of thermal denaturation.

Supplementary Figures S11-S12.

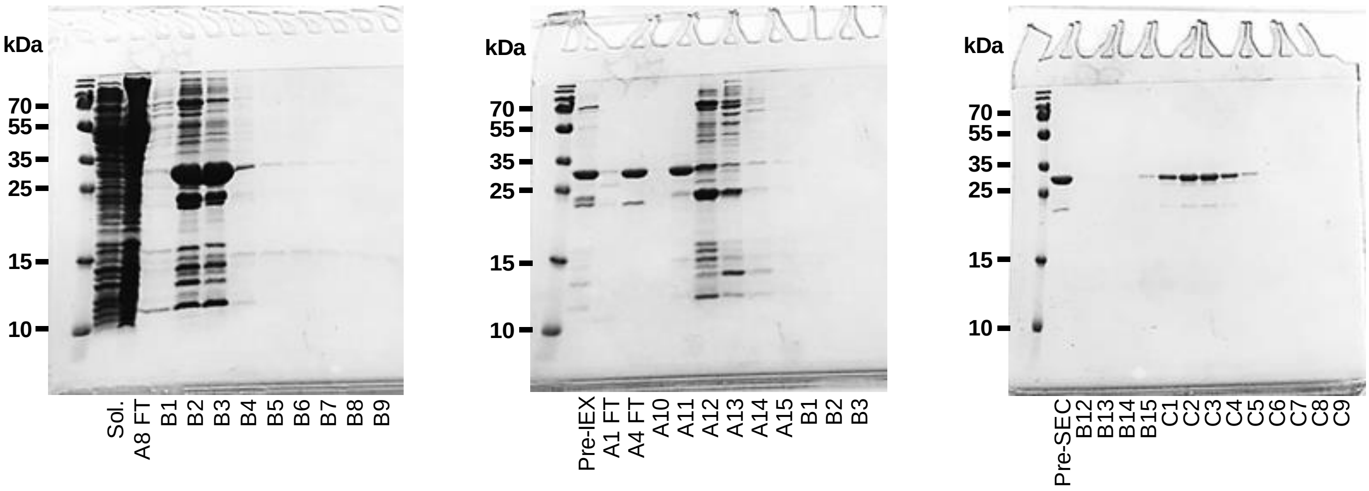

Supplementary Figure S11. Ag85b purification. Uncropped SDS-PAGE gel images of Ag85b nickel affinity (left), ion exchange (centre) and size exclusion (right) chromatography. Elution fractions only are shown in Figure 1a. Unrelated lanes have been removed. Sol., soluble lysate; FT, flow-through; pre-IEX, pre-ion exchange; pre-SEC, Pre-size exclusion.

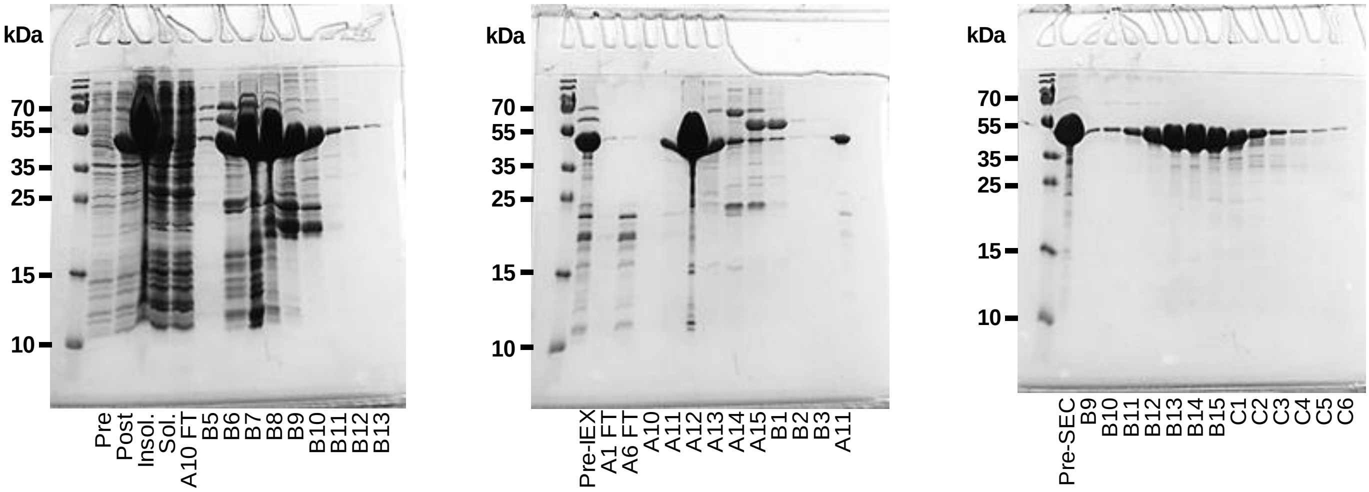

Supplementary Figure S12. Sbi III-IV-Ag85b purification. Uncropped SDS-PAGE gel images of Sbi III-IV-Ag85b nickel affinity (left), ion exchange (centre) and size exclusion (right) chromatography. Elution fractions only are shown in Figure 1b. Pre, pre-induction; post, post-induction; insol., insoluble pellet; sol., soluble lysate; FT, flow-through; pre-IEX, pre-ion exchange; pre-SEC, Pre-size exclusion.

Supplementary Figure S13.

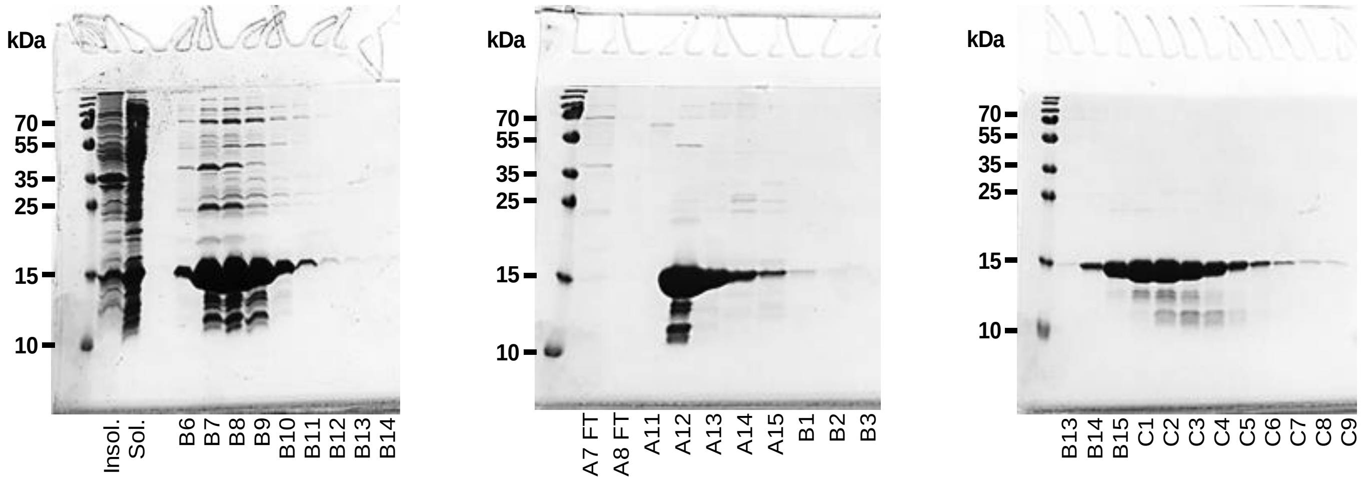

Supplementary Figure S13. Sbi III-IV purification. Uncropped SDS-PAGE gel images of Sbi III-IV nickel affinity (left), ion exchange (centre) and size exclusion (right) chromatography. Elution fractions only are shown in Figure 1c. Unrelated lanes have been removed. Insol., insoluble pellet; sol., soluble lysate; FT, flow-through.

Supplementary Figure S14

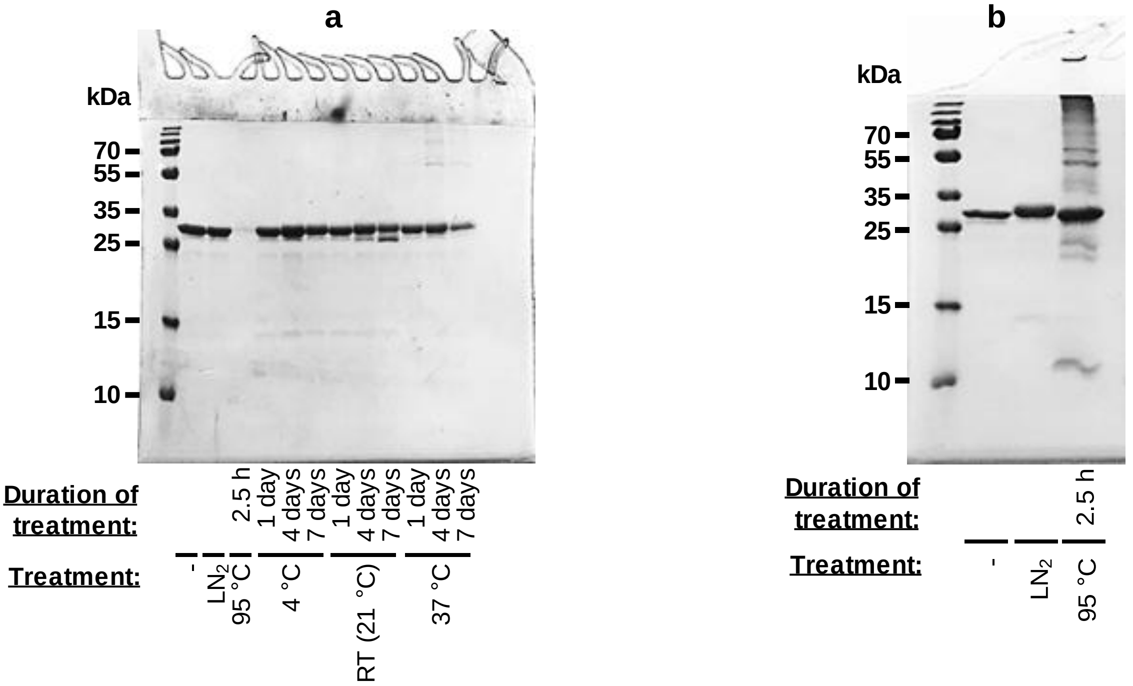

Supplementary Figure S14. Analysis of temperature-treated Ag85b. Uncropped SDS-PAGE gel images of Ag85b following the temperature treatments shown. Figure 2c shows the lane corresponding to 2.5 h at 95°C from Figure S14b merged with Figure S14a. Unrelated lanes have been removed. LN_2; liquid nitrogen.

Supplementary Figures S15-S16.

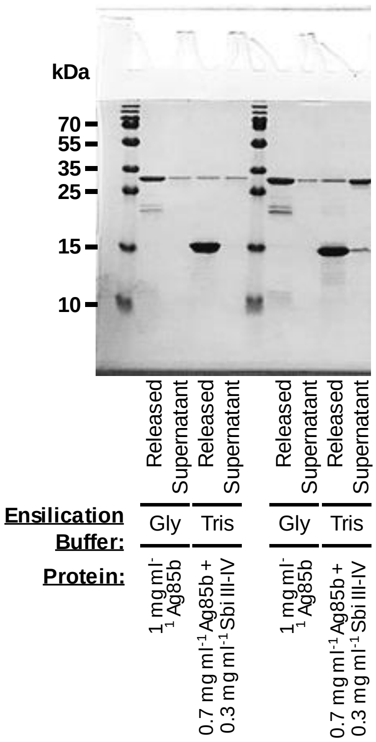

Supplementary Figure S15. Analysis of Ag85b ensilication and release. Uncropped SDS-PAGE gel image showing the composition of the released solution and unensilicated supernatant following ensilication of Ag85b in the glycine buffer or presence of Sbi III-IV as shown in Figure 2e. A larger volume of sample was loaded to the wells on the right. Unrelated lanes have been removed.

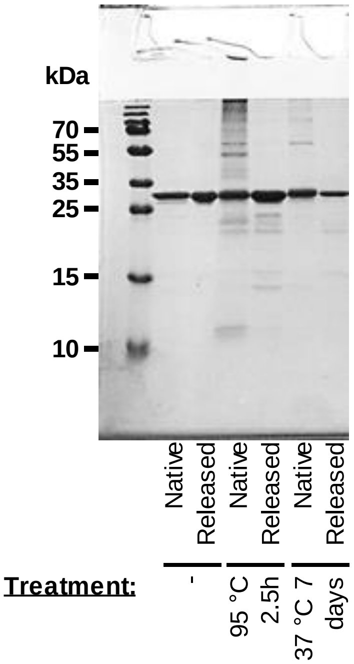

Supplementary Figure S16. Analysis of temperature-treated native and released Ag85b. Uncropped SDS-PAGE gel image showing native and released Ag85b following the temperature treatments indicated, as shown in Figure 2g. Unrelated lanes have been removed.

Supplementary Figures S17-S18.

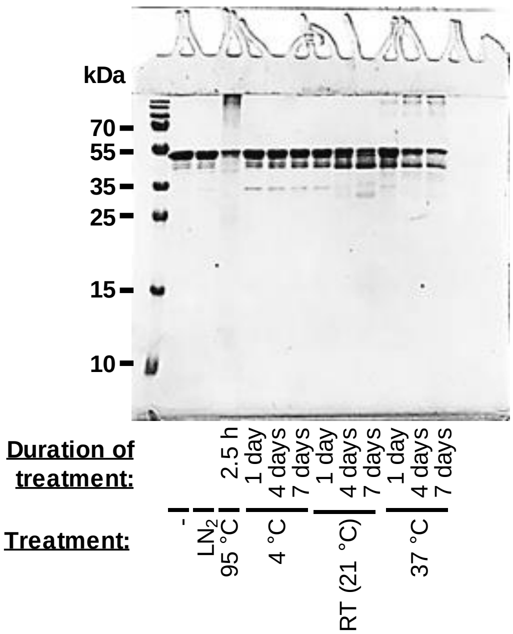

Supplementary Figure S17. Analysis of temperature-treated Sbi III-IV-Ag85b. Uncropped SDS-PAGE gel image of Sbi III-IV-Ag85b following the temperature treatments indicated, as shown in Figure 3c. LN_2; liquid nitrogen.

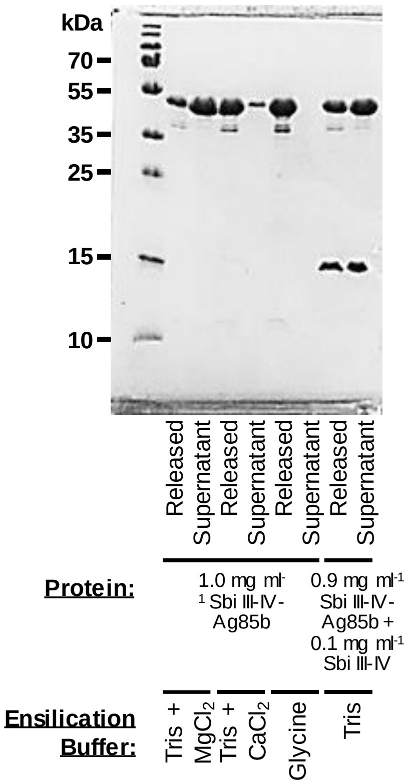

Supplementary Figure S18. Analysis of Sbi III-IV-Ag85b ensilication and release. Uncropped SDS-PAGE gel image showing the composition of the released solution and unensilicated supernatant following ensilication of Sbi III-IV-Ag85b under the conditions indicated, as shown in Figure 3f. Unrelated lanes have been removed

Supplementary Figure S19.

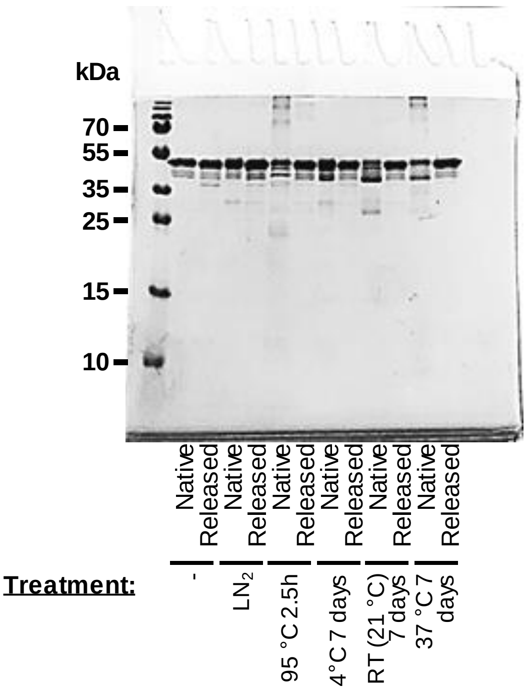

Supplementary Figure S19. Analysis of temperature-treated native and released Sbi III-IV-Ag85b. Uncropped SDS-PAGE gel image showing native and released Sbi III-IV-Ag85b following the temperature treatments indicated, as shown in Figure 3k.

Supplementary Figure S20.

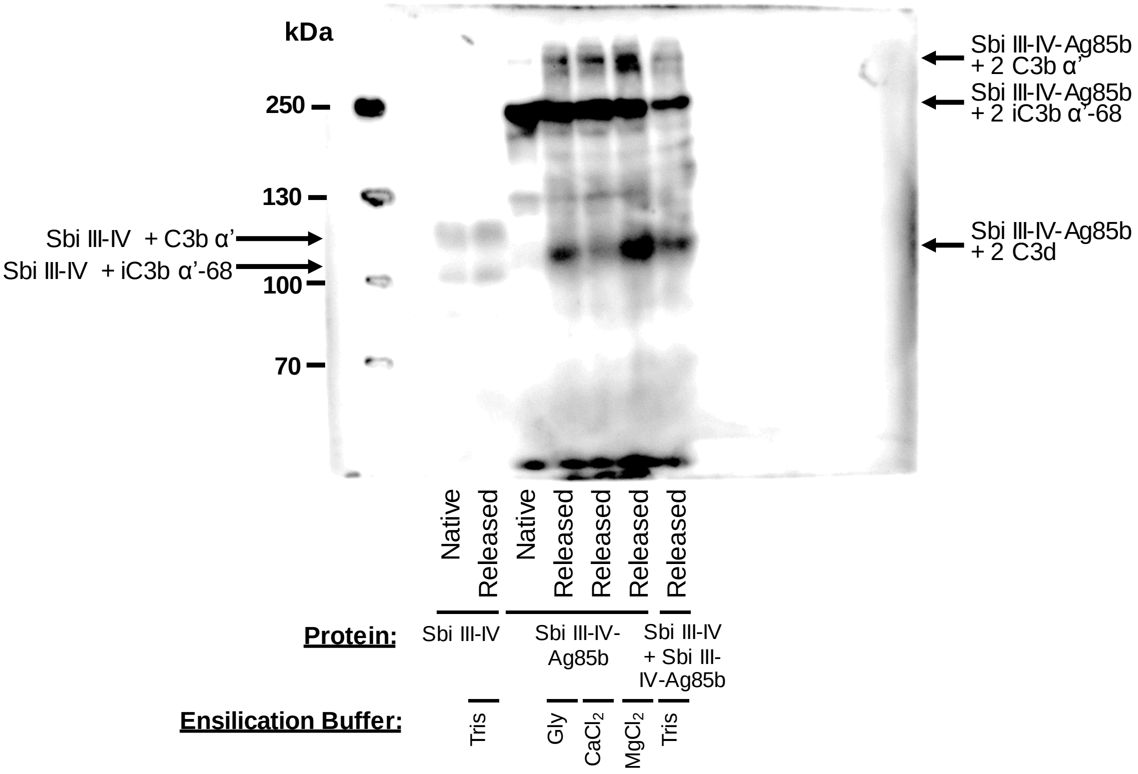

Supplementary Figure S20. Analysis of C3 activation mediated by native and released Sbi III-IV and Sbi III-IV-Ag85b. Uncropped anti-Sbi western blot image showing deposition of C3 activation products mediated by native as well as released Sbi III-IV and Sbi III-IV-Ag85b following ensilication in the buffers shown and incubation with normal human serum. Figure 3g shows the molecular weight marker merged with the Sbi III-IV-Ag85b section of the blot while Figure 4g shows the Sbi III-IV section of the blot.

Supplementary Figures S21-S22.

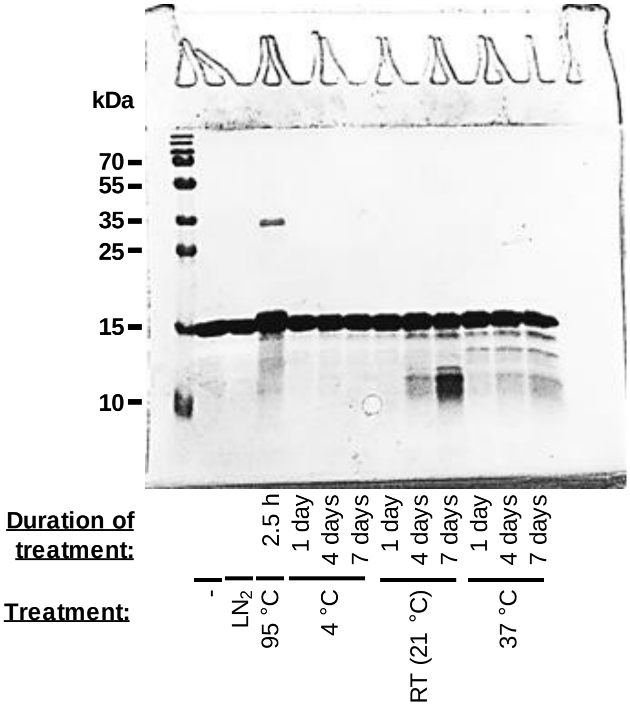

Supplementary Figure S21. Analysis of temperature-treated Sbi III-IV. Uncropped SDS-PAGE gel image of Sbi III-IV following the temperature treatments indicated, as shown in Figure 4c. LN_2; liquid nitrogen.

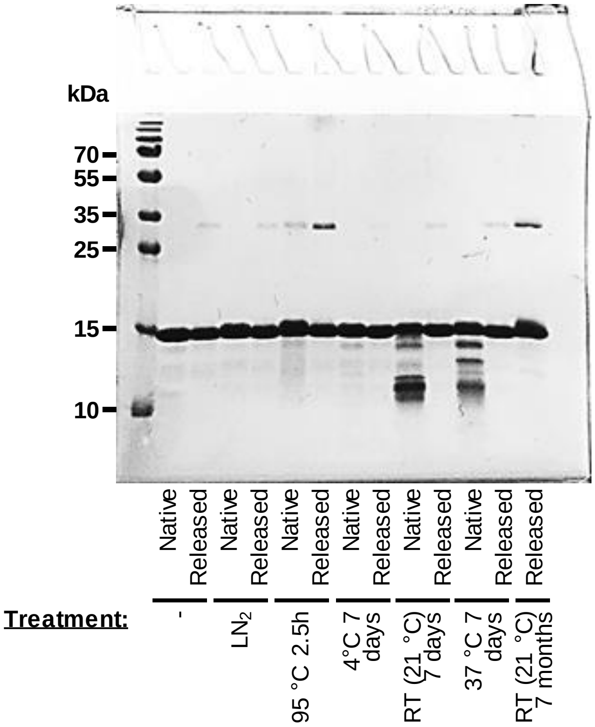

Supplementary Figure S22. Analysis of temperature-treated native and released Sbi III-IV. Uncropped SDS-PAGE gel image showing native and released Sbi III-IV following the temperature treatments indicated, as shown in Figure 4i.

Supplementary Tables S1-S3.

Supplementary Table S1. Physiochemical properties of the Ag85b, Sbi III-IV-Ag85b and Sbi III-IV protein constructs. Theoretical isoelectric points (pI) were computed using the Expasy Protparam tool.

Protein	Number of residues	MW (Da)	pI	Yield (mg L^{-1})
**Ag85b**	304	32861.61	5.65	1.6
**Sbi III-IV-Ag85b**	457	49763.14	6.53	2.7
**Sbi III-IV**	129	14828.57	9.27	2.0

Supplementary Table S2. Concentrations of protein present in the released solution, unensilicated supernatant and ensilication wash following Ag85b ensilication under two different conditions. Protein concentration was measured using a BCA assay.

Condition		Protein concentration (mg ml^{-1})		
**Protein**	**Ensilication buffer**	**Released**	**Supernatant**	**Wash**
1 mg ml^{-1} Ag85b	50 mM Glycine pH 7.4	0.65	0.16	0.02
0.7 mg ml^{-1} Ag85b + 0.3 mg ml^{-1} Sbi III-IV	50 mM Tris pH 7	0.46	0.42	0.01

Supplementary Table S3. Concentrations of protein present in the released solution, unensilicated supernatant and ensilication wash following Sbi III-IV-Ag85b ensilication under four different conditions. Protein concentration was measured using a BCA assay.

Condition		Protein concentration (mg ml^{-1})		
**Protein**	**Ensilication buffer**	**Released**	**Supernatant**	**Wash**
1 mg ml^{-1} Sbi III-IV-Ag85b	50 mM Tris, 20 mM $MgCl_2$ pH 7	0.13	0.45	0.00
	50 mM Tris, 20 mM $CaCl_2$ pH 7	0.45	0.04	0.00
	50 mM Glycine pH 7.4	0.60	0.00	0.00
0.9 mg ml^{-1} Sbi III-IV-Ag85b + 0.1 mg ml^{-1} Sbi III-IV	50 mM Tris pH 7	0.32	0.48	0.01
